# Quality Assessment of Surgical Disc Samples Discriminates Human Annulus Fibrosus and Nucleus Pulposus on Tissue and Molecular Level

**DOI:** 10.3390/ijms19061761

**Published:** 2018-06-13

**Authors:** Ann-Kathrin Schubert, Jeske J. Smink, Mirko Arp, Jochen Ringe, Aldemar A. Hegewald, Michael Sittinger

**Affiliations:** 1 Charité-Universitätsmedizin Berlin, Corporate Member of Freie Universität Berlin, Humboldt-Universität zu Berlin and Berlin Institute of Health, Tissue Engineering Laboratory and Berlin-Brandenburg Center for Regenerative Therapies, 13353 Berlin, Germany; jochen.ringe@charite.de (J.R.); michael.sittinger@charite.de (M.S.); 2 CO.DON AG, 14513 Teltow, Germany; jsmink@gmx.de; 3 Department of Neurosurgery, University Medical Center Mannheim, Heidelberg University, 68167 Mannheim, Germany; Mirko.Arp@umm.de (M.A.); aldemar.hegewald@medma.uni-heidelberg.de (A.A.H.); 4 Department of Neurosurgery and Spine Surgery, Helios Baltic Sea Hospital Damp, 24351 Damp, Germany

**Keywords:** annulus fibrosus, nucleus pulpous, microarray analysis, marker, quality assessment

## Abstract

A discrimination of the highly specialised annulus fibrosus (AF) and nucleus pulposus (NP) cells in the mature human intervertebral disc (IVD) is thus far still not possible in a reliable way. The aim of this study was to identify molecular markers that distinguish AF and NP cells in human disc tissue using microarray analysis as a screening tool. AF and NP samples were obtained from 28 cervical discs. First, all samples underwent quality sorting using two novel scoring systems for small-sized disc tissue samples including macroscopic, haptic and histological evaluation. Subsequently, samples with clear disc characteristics of either AF or NP that were free from impurities of foreign tissue (IVD score) and with low signs of disc degeneration on cellular level (DD score) were selected for GeneChip analysis (HGU1332P). The 11 AF and 9 NP samples showed distinctly different genome-wide transcriptomes. The majority of differentially expressed genes (DEGs) could be specifically assigned to the AF, whereas no DEG was exclusively expressed in the NP. Nevertheless, we identified 11 novel marker genes that clearly distinguished AF and NP, as confirmed by quantitative gene expression analysis. The novel established scoring systems and molecular markers showed the identity of AF and NP in disc starting material and are thus of great importance in the quality assurance of cell-based therapeutics in regenerative treatment of disc degeneration.

## 1. Introduction

Back pain is a major problem in today’s society. One cause is the degeneration of the intervertebral disc, which is a naturally occurring phenomenon in aging humans. The disc is a cartilage-like tissue between the vertebrae that enables mobility of the spinal unit due to its specific structural composition and matrix organisation. The cells in the disc are highly specialised cells that produce and thereby maintain the disc matrix. However, the synthesis and degradation of extracellular matrix (ECM) molecules becomes imbalanced with time. This results in a loss of disc matrix and a decrease in cell number [1,2], causing the disc degenerative disease (DDD). Recent developments have used regenerative therapies to treat DDD. The implantation of cells is one option and is classified as cell-based therapy. Cell therapies aim to repopulate the damaged disc with cells and thus structurally and functionally restore the disc’s properties [3]. To be able to repair the disc, it is essential to understand the building material of the tissue, the disc cells. Furthermore, the patient’s safety—and thus the quality of the cell-based product—is of highest priority. This requires the clear identification of the cells before implantation, to make sure the implanted cells are indeed disc cells and retain their specific characteristics. Hence, it is important to characterise disc cells and to show the identity of these cells for quality assessment in disc cell therapy.

The disc does not consist of one tissue, but consists of distinct compartments, the annulus fibrosus (AF) and nucleus pulposus (NP), containing different cell types, the AF and NP cells. Hence, it is important to be able to discriminate between these two cell types. The AF and NP originate from different lineages during embryonic development and have a different macroscopic appearance [4]. The AF is a stiff ring-shaped multilayer of lamellae with aligned collagen fibres, whereas the NP appears spongy due to its high hydration and random organisation of collagen fibres [1]. Due to their different ECM content and organisation, AF and NP have a different function during spinal movement. The central NP absorbs the shock while the surrounding AF distributes the mechanical shock [5]. Although these two disc compartments are obviously different in morphology and function, AF and NP cells are thus far not distinguishable at the molecular level [6,7].

To describe the diversity of disc cells, recent studies have applied microarray analysis on rat, canine and bovine disc tissue [8,9,10,11]. However, cell populations in these non-human discs are considerably different from human disc cells [12]. In the NP compartment, another cell type is targeted than in humans, the notochordal (NC) cells. In humans, the NC cells are predominantly present during NP development, diminish with the age and get replaced in mature NP, whereas NC cells remain in animals through adolescence [13,14]. Due to the discussion about the presence of NC cells in mature human discs, it has been unclear for a long time whether the results obtained from animal studies were transferable to humans [15,16]. Minogue et al. demonstrated clear interspecies variations in bovine and human disc cell phenotypes [10]. As the validity of markers for non-human disc cells was not shown for human disc cells, the focus changed towards using human cells instead of animal cells in the recent years. Nevertheless, most investigations address the molecular changes in disc degeneration to understand and challenge the disease rather than the discrimination of AF and NP cells [17,18,19,20,21]. In addition, the NP has initially been of more interest in disc tissue engineering, as this compartment is affected first in disc degeneration [1]. Therefore, there is only consensus about the phenotype of young and healthy NP cells. To show the NP phenotype, the following markers are recommended: *aggrecan* (*ACAN*), *carbonic anhydrase 12* (*CA12*), *collagen type II* (*COL2A1*), *hypoxia inducible factor 1 alpha subunit* (*HIF1A*), *keratin 18* and *19* (*KRT18*, *-19*), *solute carrier family 2 member 1* (*SLC2A1*), *sonic hedgehog* (*SHH*) and *T brachyury transcription factor* (*T*) [22]. The developmental genes *SHH* and *T* are, however, not expressed in mature human NP cells, whereas the other recommended marker genes are also expressed in AF and articular cartilage [10,23,24]. Therefore, these are rather functional markers to characterise cells from the cartilaginous lineage, but are not suitable to distinguish AF and NP cells [22]. Hence, there is still a need for human AF and NP markers.

The aim of this study was to identify molecular markers that discriminate human AF and NP cells. As the distinct AF and NP transcriptomes were the basis for marker identification we performed sample quality sorting to only use AF and NP tissue samples with clear characteristics for microarray analysis. Therefore, we used a novel quality assessment system, which included macroscopic, haptic and histological sample evaluation. We utilised genome-wide transcriptome analysis as a screening tool for AF and NP markers that are differentially expressed in AF and NP tissue. Furthermore, we performed quantitative gene expression analysis of the identified markers to proof their distinct expression in AF and NP tissue obtained from human disc samples.

## 2. Results

### 2.1. Quality Assessment of AF and NP Tissue Samples with a Novel Scoring System: IVD and DD Score

**Figure 1:**
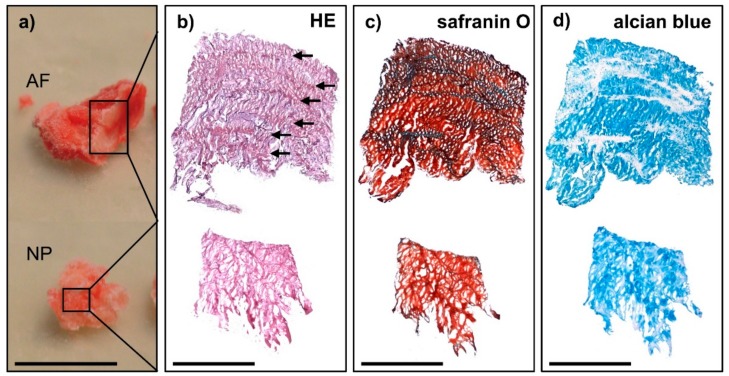
Exemplary small-sized tissue samples that showed the appropriate tissue type of either annulus fibrosus (AF) or nucleus pulposus (NP) according to IVD scoring. (**a**) Macroscopic and (**b**–**d**) histological evaluation with (**b**) haematoxylin-eosin (HE) staining (fibres typical for AF are indicated by arrows), (**c**) safranin O staining (proteoglycans in red) and (**d**) alcian blue staining (proteoglycans in blue). Scale bar in (**a**) 10 mm and in (**b**–**d**) 1 mm.

**Figure 2:**
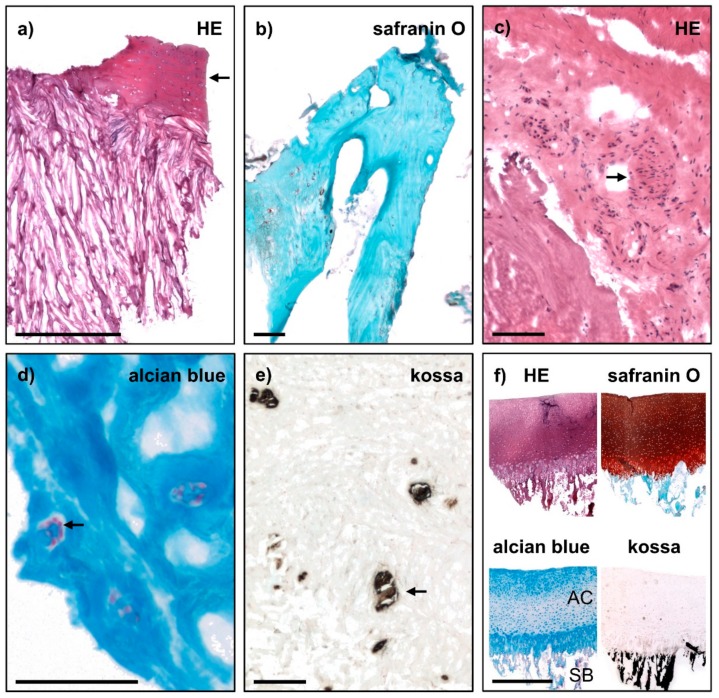
Histology of exemplary samples that showed (**a**–**c**) impurities and (**d**–**e**) severe signs of degeneration on cellular level according to IVD and DD scoring. (**a**) Cartilage endplate, (**b**) bone, (**c**) unusual cell accumulation, (**d**) clustered cells and (**e**) calcium deposits (all indicated by arrow). (**f**) Staining controls of porcine articular cartilage (AC) with adjacent subchondral bone (SB) for (**a**,**c**) haematoxylin-eosin (HE) staining, (**b**) safranin O staining (cartilage in red, bone in cyan), (**d**) alcian blue staining (cell nuclei in pink, proteoglycans in blue) and (**e**) von Kossa staining (calcification and SB in black, cell nuclei in pink). Scale bar in (**a**,**f**) 1 mm and in (**b**–**e**) 0.1 mm.

For the identification of AF- and NP-specific markers, samples were needed that were of clear AF and NP origin, not being impurified by other cell types. In addition, as the focus was not the degenerative state of the disc tissue, or the identification of degeneration markers, it was important to evaluate the level of degeneration. Therefore, all samples included in this study first underwent a novel quality assessment system developed by us: the intervertebral disc (IVD) score and the disc degeneration (DD) score. This evaluation system was applied to small-sized tissue samples obtained from the AF and NP compartments during surgical removal of human disc tissue. The IVD score assessed individual characteristics typical for either AF or NP and sample purity (Table 1). To classify the grade of disc degeneration on the cellular level, we used the DD score (Table 2). The novel scores were adapted from literature [25,26], modified for small-sized tissue samples and additionally included criteria according to the findings in macroscopic, haptic and histological evaluation (Figure 1 and Figure 2). The results of both IVD scoring and DD scoring are shown in Table 3.

The IVD score included defined AF and NP characteristics in morphological organisation and haptic appearance (Table 1 and Figure 1). In macroscopic evaluation, AF tissue was dense and showed the typical concentric sheets of fibres in several layers, whereas NP tissue was spongy and had a soft and loose appearance (Figure 1a). The specific tissue type was further examined using histological staining. In staining for gross morphology, AF samples showed a clearly defined half-ring shaped arrangement of fibres, whereas NP samples had a well-organised extracellular matrix without fibres (Figure 1b,c). Furthermore, safranin O and alcian blue staining were used to score the appropriate expression and distribution of proteoglycans in the ECM (Figure 1c,d). Less intense or irregular staining was a sign for a less defined AF or NP character.

For the purpose of this study, it was further necessary to exclude the evidence of sample impurities. In most samples, other tissue structures were not visible either in macroscopic, haptic or histological analysis. However, some samples additionally showed tissue structures like cartilage endplate (CEP) (Figure 2a) or lamellar bone (Figure 2b) that were comparable to articular cartilage (AC) and subchondral bone (SB) in the staining control (Figure 2f), respectively. An impurity with CEP was more often found in NP samples, whereas bone impurity was restricted to AF samples. Furthermore, some AF samples showed cell accumulations usually not present in the disc (Figure 2c) or parts of ligamentum flavum (not shown).

In summary, 17/28 AF (61%) and 16/28 NP (57%) passed IVD scoring by reaching a score of 6 without any scoring item scored 0 (Table 3). These samples were classified as clear AF and clear NP, meaning a sample with defined characteristics of either AF or NP not containing the other disc tissue or other foreign tissue. All other samples were undefined and were thus classified as unknown samples.

Besides selecting the correct tissue type, it was of major importance to identify samples with proceeded disc degeneration, as disc degeneration impedes the AF and NP transcriptome in microarray analysis [17,28]. To grade disc degeneration on the cellular level, we use the DD score (Table 2). A greyish and sclerotic appearance in macroscopic and haptic evaluation, respectively, indicated a proceeded degeneration in the sample. Furthermore, enlarged cells and increased cellularity have been described for degenerated discs compared to healthy discs. To visualise these signs of degeneration, alcian blue staining was very helpful, as glycosaminoglycan (GAG) synthesis is also enhanced in cells of the degenerated disc. Predominantly NP samples showed an increased cellularity, as cells arranged in clusters or chondroid nests (Figure 2d). In contrast, calcification, another a sign of disc degeneration, was more often observed in AF samples (Figure 2e).

In total, 38% of all samples reached a DD score of 3 or scored 2 in any scoring item (Table 3). These samples had severe signs of degeneration on the cellular level and were thus excluded from microarray analysis. All other samples were classified as samples with mild degeneration, because all discs showed degenerative changes on the whole disc level in magnetic resonance imaging (MRI), and almost every disc is affected by degeneration in humans older than 50 [29].

### 2.2. Correlation of Scoring Outcome with Donor Age and MRI Grade of Degeneration

Spearman’s correlation analysis was performed to determine the relationship between outcome of IVD scoring and DD scoring with either donor age or MRI disc degeneration grade in both AF and NP samples (Table 4). The outcome of IVD scoring and donor age showed a moderate negative correlation for NP samples, meaning that the older the donor the lower the IVD scoring value. Hence, discs from older donors were more likely to have an undefined NP containing foreign tissue. For all other comparisons, there was no correlation.

### 2.3. Genome-Wide Expression Analysis Revealed Distinct AF and NP Transcriptomes

For microarray analysis, only AF and NP samples were considered that passed the new quality assessment system. IVD scoring and DD scoring were applied subsequently to only use those samples with clear disc characteristics and low degeneration on cellular level, respectively. Hence, 15 AF and 11 NP samples had a clear character of either AF or NP (IVD score), and showed mild degeneration (DD score) (Table 3). However, only 11 AF and 9 NP samples were derived from different donors (*n* = 13) and thus used for microarray analysis. The mean age was 53 years (range 36–76 years, female/male ratio 6/7).

**Figure 3:**
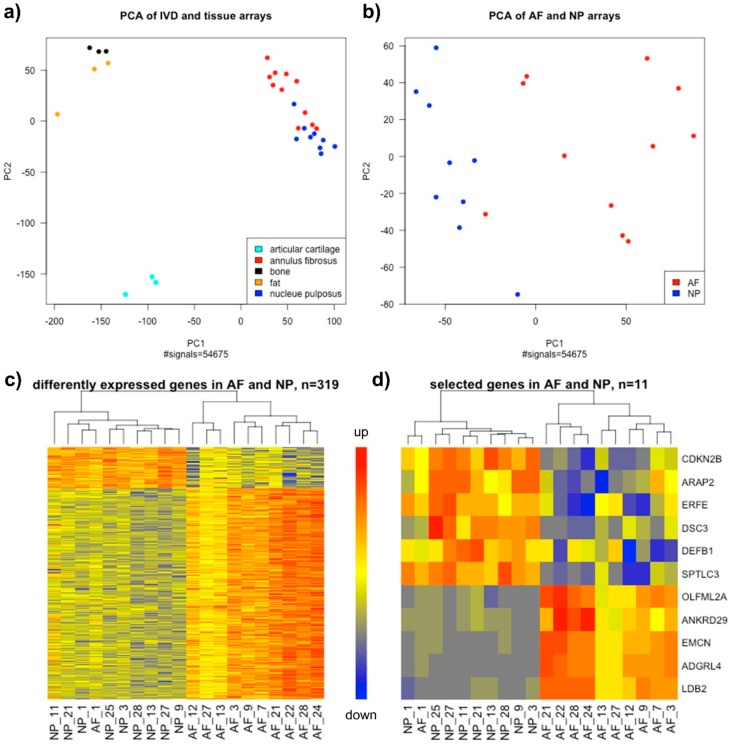
Clustering of microarray data obtained from annulus fibrosus (AF) and nucleus pulposus (NP) tissues samples. (**a**,**b**) Principle component analysis (PCA) shows statistical clustering of total genome-wide transcriptome of different tissues in (**a**) intervertebral disc (IVD): AF (red, *n* = 11), NP (dark blue, *n* = 9), and other mesenchymal tissues (each *n* = 3): articular cartilage (cyan), cancellous bone (black) and fat (orange), and in (**b**) only AF (red, *n* = 11) and NP (dark blue, *n* = 9). (**c**,**d**) Heatmaps showing hierarchical clustering of differentially expressed genes (DEGs) in AF and NP samples regarding (**c**) all 319 DEGs and (**d**) 11 selected marker genes (red up-regulated and blue down-regulated). Graphs were created with R (after RMA-normalisation of the samples presented in the individual graphs).

To show the difference of the AF and NP transcriptome to other mesenchymal tissues, we additionally included previously published microarray data from human fat tissue [30] and articular knee cartilage [31] and unpublished microarray data from human cancellous bone (in preparation). Array data were generated with the same protocol and were only used for principle component analysis (PCA). PCA of all tissue arrays showed three distinct groups: (1) AF and NP, (2) articular cartilage, and (3) bone and fat (Figure 3a). Although AF and NP were clearly separated from all other mesenchymal tissues, the two disc populations were arranged very closely. Nevertheless, PCA of only AF and NP arrays showed a distinct separation of the two disc tissue types (Figure 3b). Hence, we obtained distinct AF and NP transcriptomes that were used as basis for the subsequent marker screening.

### 2.4. Screening for Marker Genes Differentially Expressed in AF and NP Tissue

In this study, microarray data based on 11 AF and 9 NP samples from individual donors. The big sample size made the microarray data statistically reliable and thus enabled another screening tool for differentially expressed genes (DEGs). In addition to the fold-change (FC), we could use the percentage of change between AF and NP (change%) to show a relevant gene regulation in either AF or NP. Thereby, we excluded genes that already failed in testing for unique expression in disc cells, e.g., *cartilage oligomeric matrix protein* (*COMP*), *KRT19* or *tenomodulin* (*TNMD*) [8,32]. In total, 319 genes were differentially expressed between AF and NP with a fold-change larger than 2 in at least 80% of all pairwise comparisons between all AF and NP samples. The number of DEGs comprised 1.7% of the total transcriptome. Hierarchical clustering of all AF and NP arrays according to the DEG expression profile resulted in two main groups, clearly separating AF from NP samples (Figure 3c). 267 DEGs were up-regulated in AF compared to NP and 52 DEGs were higher expressed in NP compared to AF. The highest fold-change was 124.7 ± 7.5 and 5.2 ± 0.4 in AF and in NP, respectively. Therefore, we obtained two gene lists containing marker candidates for subsequent marker identification (Supplement Tables S1 and S2).

For marker identification, the gene lists were ranked according to FC, because the markers should have a clearly different expression in AF and NP. The top 25 ranked DEGs of each list were taken to search for gene information and published data regarding the disc or other relevant research areas in the NCBI database. Thereby, we excluded genes with connections to disc degeneration or other cartilage-related diseases, e.g., osteoarthritis, as well as genes with known ubiquitous expression in the human body or articular cartilage. From the remaining list of AF marker candidates, we selected five genes that showed a high fold-change (FC ≥ 24.4, top25) with significant *p*-value (<0.05) and showed no detection in the NP (detection% = 0). These selection criteria were not completely applicable for NP marker selection, because no DEG was exclusively detected in the NP. Therefore, we chose six genes with clear up-regulation in the NP (FC ≥ 3.0, top16) and additionally stable signal detection in the NP (signal mean).

Finally, the selected set of 11 markers included the genes encoding cyclin dependent kinase inhibitor 2B (*CDKN2B*), ArfGAP with RhoGAP domain, ankyrin repeat and PH domain 2 (*ARAP2*), erythroferrin (*ERFE*), desmocollin 3 (*DSC3*), defensin beta 1 (*DEFB1*), serine palmitoyltransferase long chain base subunit 3 (*SPTLC3*) (*NP markers*) and olfactomedin-like 2A (*OLFML2A*), ankyrin repeat domain 29 (*ANKRD29*), endomucin (*EMCN*), adhesion G protein-coupled receptor L4 (*ADGRL4*) and LIM domain binding 2 (*LDB2*) (*AF markers*). The NP marker genes were clearly up-regulated in NP compared to AF, whereas the AF markers were exclusively expressed in AF samples (Figure 3d). These markers represented different biological functions, e.g., signal transduction, cell adhesion, cell cycle or biosynthetic processes.

### 2.5. AF and NP Marker Expression in Clear and Unknown Disc Tissue Samples

**Figure 4:**
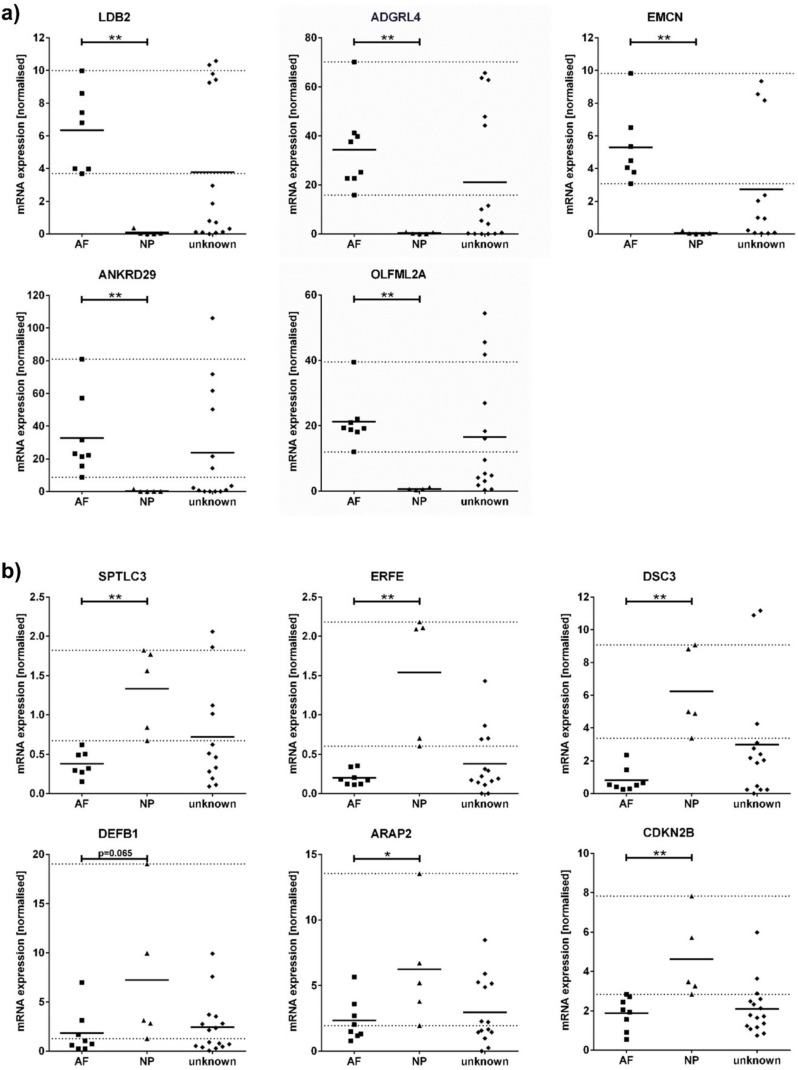
Quantitative gene expression analysis of (**a**) AF and (**b**) NP markers in samples with clear characteristics of AF (squares, *n* = 9) or NP (triangles, *n* = 5), and in samples with unknown tissue characteristics (diamonds, *n* = 16, 1:1 original resection site AF or NP) according to IVD scoring. Results are shown as mean (horizontal bar) with an individual data point for each sample (gene expression level with normalisation to reference genes ATP5F1B and RPL13A). Please note that individual data points are missing as for some samples there was not enough RNA available to perform all measurements. Dashed lines present highest and lowest expression level of each marker in the corresponding tissue (range). Significance * *p* < 0.05, ** *p* < 0.01 AF compared to NP.

The set of 11 identified AF and NP markers was further analysed using quantitative gene expression analysis of 30 test samples obtained from the AF and NP compartment during surgical resection of the human discs (Table 3). The test samples were grouped according to their outcome in IVD scoring. Groups thus represented test samples with either clear characteristics (IVD score > 6 and no scoring item graded 0) of AF (*n* = 9) and NP (*n* = 5) tissue or with undefined characteristics (unknown) resected from AF and NP (*n* = 16) but not determined as AF or NP, respectively (IVD score ≤ 6 or any scoring item graded 0). The gene expression data of AF and NP markers are illustrated in Figure 4a,b, respectively.

AF and NP marker gene expression was different between the clear AF and NP samples, confirming the microarray results. All AF markers were significantly higher expressed in AF compared to NP (Figure 4a). Furthermore, the AF markers showed a high expression level in all AF samples, whereas no AF marker was detected in the NP samples. By contrast, the NP markers were expressed in both NP and AF (Figure 4b). Gene expression level of NP markers *DEFB1* and *ARAP2* in 3 AF samples overlapped with the NP markers expression range defined by the clear NP samples (dashed lines). Nevertheless, gene expression of *SPTLC3*, *ERFE*, *DSC3*, *CDKN2B* and *ARAP2* was significantly higher in NP compared to AF.

Furthermore, the novel markers were applied to 16 test samples with undefined (unknown) disc characteristics to classify their origin in either AF or NP compartment of the disc. For this, we used the gene expression range of the novel AF and NP markers defined by samples with clear AF and NP characteristics, respectively (dashed lines in Figure 4). The individual marker expression profile of each unknown sample is shown in Table 5. 5 unknown samples expressed all AF markers and lacked NP marker expression. 3 unknown samples showed a positive NP marker profile and no expression of any AF marker. However, only 2 of these samples were originally obtained from the NP. The third sample was resected from the AF compartment. The contrary marker expression profile in this sample supported the missing AF appearance in IVD scoring and thus revealed the false initial assignment to the AF during resection. The other 8 unknown samples matched neither the AF nor the NP marker profile and thus tissue classification of these test samples remained undefined. Therefore, the individual AF and NP marker expression profiles confirmed the correct tissue origin of 5/8 AF and 2/8 NP surgical disc samples.

## 3. Discussion

In this study, we implemented a novel quality assessment system for small-sized tissue samples from either AF or NP to classify their specific tissue character and disc degeneration grade on a cellular level using the IVD score and DD score, respectively. We used this evaluation system to select accurately discriminated AF and NP tissue samples with low levels of degeneration for microarray analysis. Using these specifically selected tissue samples, we were able to generate a very specific data set of the AF and NP transcriptome. Genome-wide transcriptome analysis served as a screening tool to identify new molecular marker genes that discriminate AF and NP. The suitability of the novel AF and NP markers was verified in quantitative gene expression analysis of AF and NP tissue samples. Finally, the novel markers were suitable to identify AF and NP among samples with unknown tissue character.

The selection of starting material for microarray analysis was a crucial point in this study. The samples used in this study were derived from human cervical discs and were separated during surgical resection. As AF and NP merge during disc degeneration, these two compartments get less distinguishable by eye with progressed degeneration. Disc degeneration is lower in cervical compared to lumbar discs [33]. Therefore, the choice of cervical discs was required to facilitate an intra-surgical separation of AF and NP. In addition, all samples underwent macroscopic, haptic and histological examination to confirm the correct origin from either AF or NP compartment of the disc. Most of the samples showed typical characteristics of AF and NP tissue as described by others in studies on human disc tissue and thus suggested a clear separation of AF and NP [34,35]. However, some samples showed mixed characteristics of AF and NP or contained parts from the surrounding tissue, like cartilage endplate or bone. Furthermore, cell accumulations indicated another type of impurity in these samples as AF and NP have a very low cellularity [36]. Any impurity, even though present in only a small part of the sample, could interfere with the analysis, and microarray data would not present a distinct AF and NP transcriptome. In addition, a similar degenerative state of the samples used for microarray analysis was important, as disc degeneration impedes AF and NP transcriptome data [17,28]. Therefore, systematic quality assessment of all samples was necessary to determine the tissue origin and purity, as well as to evaluate the degenerative state.

To address the sample quality of AF and NP tissue necessary for this study, no evaluation system was available in the literature. Existing histological classification systems of disc tissue samples focus on the grading of disc degeneration [25,26,37,38] rather than discrimination of AF and NP tissue samples. Furthermore, these scoring systems require whole disc specimen or samples with an intact transition zone between AF and NP. In this study, AF and NP were already separated and only represented a part of the total disc. Therefore, we developed a novel evaluation system to classify the tissue character (IVD score) and grade of disc degeneration (DD score), which allowed quality sorting of the small-sized disc tissue samples as used in this study.

The IVD scoring included the evaluation of characteristics known for AF and NP tissue. The most reliable criterion to discriminate AF and NP was the structural organisation of the ECM, especially the presence or absence of fibres. The AF was arranged in multilayers of half-ring-shaped annular fibres, whereas the NP had a well-organised ECM that did not contain fibres. To address this scoring item, we used HE and safranin O staining, whereby the fibres were better visualised in safranin O staining. As adjacent fibres arrange orthogonally in the AF, the staining for cartilaginous structures and GAGs appeared to be differently intense in the various layers. The counterstaining with fast-green further highlighted the difference between the cross-sectioned and in-plane lamellae in the AF [39,40]. AF and NP samples were scored separately with pre-specification of the sampling location, AF or NP. Otherwise, samples taken from the AF with a bad collagen orientation could be misinterpreted to be NP, and NP samples with an AF impurity could falsely be interpreted as AF. As the IVD scoring also addressed the evidence of sample impurities from other tissues, we could further exclude samples containing structures not belonging to either AF or NP. These impurities included bone, cartilage or tendon, which intruded into the samples from the surrounding tissue of the disc, like vertebrae, cartilage endplate or ligamentum flavum, respectively. According to their IVD score, 59% of all samples showed either a clear AF or NP character and were free from impurities with foreign tissues. These samples passed the quality sorting.

The DD score was adapted from histological classification of disc degeneration described in the literature and modified for small-sized IVD samples [25,26]. AF rupture or NP disorganisation are typical signs of disc degeneration, but were difficult to determine on small tissue samples. The presence of cell clusters or local dispositions of proteoglycans in the pericellular matrix, as well as calcification, were more suitable criteria to describe degeneration on the cellular level. According to their DD score, only 38% of all samples had severe signs of degeneration, even though all discs showed radiographic degenerative changes according to the Miyazaki classification for cervical discs [27]. These samples were excluded from microarray analysis. The outcome of DD scoring for small-sized IVD samples was independent from MRI degeneration grade and donor’s age. We could even demonstrate that some samples from older donors hardly showed signs of degeneration and the tissue structure was still defined, whereas some samples from younger donors already exhibited severe alterations. In contrast, radiographic studies of total discs showed that disc degeneration occurs in almost every disc in human subjects older than 50, and progress of disc degeneration is age dependent [25,29,41]. However, age is not a key factor in disc pathology and MRI grading does not represent molecular degeneration [42,43,44]. Although age and MRI grading are good risk predictors for disc degeneration, these should not be the absolute exclusion criteria when selecting samples for studies on disc degeneration. Furthermore, the degree of degeneration is different in the anterior and posterior region of the disc [35,45]. A proceeded degeneration might be detected in the MRI of a total disc, whereas the same disc could locally show only a low level of degeneration. Small-sized samples could thus still represent a more healthy state of the disc even though belonging to a risk group. Therefore, the DD score is valuable to identify samples with less degeneration on cellular level.

Only samples that passed both IVD and DD scoring were used for microarray analysis. Based on their IVD and DD score, 11 AF and 9 NP samples were suitable for microarray analysis. The number of samples was not equally distributed between AF and NP, as correlation analysis showed that NP samples from older donors were more likely to fail in the IVD scoring. Nevertheless, we provided a representative sample size of both AF and NP on a native tissue level that makes the array data valuable for other investigations.

To our best knowledge, this is the first study representing the native human genome-wide transcriptome of AF and NP cells on a tissue level. The native state of the tissues was preserved by RNA isolation directly from the snap-frozen tissue. There has been only one study so far using native tissue from AF, while NP was not included in that investigation [18]. Other available microarray data was obtained from cells after tissue processing, such as by enzymatic tissue digestion [10,17,19,46]. Although enzymatic cell isolation shows no effect on gene expression of a small group of genes in AF and NP of rats, it is not known whether these findings are transferable to the total transcriptome [8].

Genome-wide expression analysis of the AF and NP array data revealed distinct AF and NP transcriptomes. Principle component analysis showed a distinct separation of AF and NP samples from other mesenchymal tissues, like bone, fat and cartilage. Furthermore, we observed a clear differentiation between the selected AF and NP samples, and thus confirmed good quality sorting. Therefore, the IVD and DD scoring was a powerful tool to specifically select AF and NP samples with distinct transcriptomes. The observed difference of 1.7% of all expressed genes in the total transcriptome was, however, lower than expected. During embryogenesis, AF and NP tissue originate from different lineages and thus develop an obviously different macroscopic appearance and ECM organisation [4]. However, in adults, the disc adapts to biomechanical loading that results in reorganisation of the inner AF, resulting in a smooth transition between AF and NP [6]. We assume that in mature discs, AF and NP also converge on molecular level and thus showed less differences in the genome-wide transcriptomes of AF and NP tissue. Microarray data from other groups also show high similarity between the AF and NP transcriptome [8,46]. Nevertheless, we selected a set of novel 11 AF and NP marker genes using a specific screening for marker genes differentially expressed in AF and NP tissue, as described in the results. The AF markers (*ANKRD29*, *ADGRL4*, *EMCN*, *LDB2*, *OLFML2A*) and NP markers (*ARAP2*, *CDKN2B*, *DEFB1*, *DSC3*, *ERFE*) clearly differentiated between AF and NP in microarray analysis, meaning that these genes showed the highest and most stable regulation in AF and NP.

Besides the novel AF and NP markers, a lot of other genes related to the disc have previously been described in the literature [9,10,32,41,46,47,48,49,50,51,52,53]. A comprehensive list is shown in Supplement Table S3. Gene expression studies reported several genes with higher expression in human NP than in AF using PCR and smaller sample numbers [41,46]. However, our microarray data obtained from 11 AF and 9 NP samples could not verify a differential expression of most of these previously reported genes in AF and NP (Supplement Table S3). Interestingly, and in line with the literature, *desmocollin 2* (*DSC2*) was up-regulated in NP samples. One of our novel NP markers is *DSC3*. Furthermore, a higher expression in non-human NP is reported for *CD24*, *forkhead box F1* (*FOXF1*), *KRT8*, *KRT18*, *versican* (*VCAN*) and *SLC2A1*, whereas studies on human discs already show a similar expression level of these genes in AF and NP [9,10,24,41,52,53]. In our study, none of these genes showed an up-regulation in NP. Furthermore, *KRT19* and *sclerostin domain containing 1* (*SOSTDC1*) have once been described as promising markers for bovine and canine NP cells [9,10]. Neither gene was detected in the human disc samples of the current study. Our data thus confirmed that disc-related genes identified in animal models are not necessarily transferable to human disc tissue [7,54].

From the genes previously reported with higher expression in either AF or NP, only 5 genes were among the DEGs identified in the microarray data of this study. *SPARC like 1* (*SPARCL1*), *secreted frizzled related protein* (*SFRP2*), *serpin family F member 1* (*SERPINF1*) and *COL12A1* were up-regulated in AF, whereas *DSC2* was higher expressed in NP compared to AF. These DEGs are also regulated in either AF or NP of bovine and canine discs [9,10,41,50], whereas a different expression in human disc tissue is only reported for *SPARCL1* and *DSC2* [41,46]. However, *SPARCL1* is down-regulated during cartilage degeneration and associated with osteoarthritis [55,56]. Furthermore, none of them was an explicit on/off marker, FC was comparatively low, or the detected signal in the corresponding tissue was very low. Therefore, these DEGs were not our first choice to serve as AF and NP markers.

Although *TEK receptor tyrosine kinase* (*TEK*) was up-regulated in AF compared to NP, this gene cannot serve as a reliable marker to discriminate AF and NP. *TEK* is commonly used to identify progenitor cells with multipotency and self-renewal capacity in the disc and *TEK*+ cells were assigned to the NP [51]. Our results showed no detection of *TEK* in the NP as *TEK*+ NP cells diminish with age [57]. A *TEK* expression in human mature AF has not been described yet. We assume that progenitor cells are not restricted to the young NP, but the mature AF could also contain a subpopulation of progenitor cells throughout adolescence. These cells were not derived from the notochord, as we could not detect the developmental/notochordal cell markers *T* or *SHH* [58]. Therefore, the detection of *TEK* in AF cells raises the question for the presence of different subpopulations of AF and NP cells in the adult disc rather than its suitability to serve as discrimination marker between AF and NP.

Other genes related to the disc showed a stable signal in both AF and NP and were not suitable to discriminate specifically the AF and NP. This does not necessarily reduce their ability to describe the disc’s nature and thus could still serve as general disc markers. *HIF1A* and *CA12* are specific to cell function in the disc environment, whereas *ACAN*, *VCAN*, *biglycan* (*BGN*), *decorin* (*DCN*) and *fibromodulin* (*FMOD*) describe the ECM content [59]. As these molecules are also present in articular cartilage and other fibrocartilages [23,60], we assume that these genes have a more general function in cartilage, like adaption to the harsh local environment or maintenance of the ECM, and thus are not only dedicated to the disc.

In this study, none of the identified DEGs was exclusively detected in NP, the number of up-regulated genes was lower in NP compared to AF, and their fold-change was low. This again raises the question for a heterogeneous cell population in the mature NP as already expected by others [23,50]. In adults, there is no original NP because of the transformation of this disc compartment through life [58]. In embryonic disc development, the predominant NP cell type is originated from the notochord. In adults, these notochordal cells get replaced by other cells with no clearly identified origin [61]. Furthermore, cell migration is triggered after NP impairment [62]. The cell type infiltrating the NP is, however, not known. It was shown by others that AF cells have the ability to migrate in vitro [63,64]. From the results in this study, this was not conclusive for native tissue, because the genes up-regulated in AF were not present in NP. This could be explained either by the low degeneration grade of the disc tissue samples and thus missing trigger for migration like inflammation or wound healing, or migration of AF cells does not occur in vivo (no study known so far). Furthermore, cells could be recruited from either CEP or adjacent vertebrae [65,66]. A stem cell population found in the CEP is more migrative than AF or NP cells in vitro [67]. The CEP could thus serve either as origin or gateway of migrative cells that then first settle down in the closely located NP. Through disc degeneration, some of the cells in the NP become senescent, and the NP thus undergoes another phenotypic shift [68]. Hence, we assume various cell populations in the mature NP that are not only different from AF cells but also among each other. Therefore, no single and exclusive marker gene might exist for the adult NP to discriminate it from AF and a specific marker set is needed.

Using the novel AF and NP markers in quantitative gene expression analysis, we could clearly discriminate AF and NP tissue samples, and thus verified the microarray results. The AF marker genes *ANKRD29*, *ADGRL4*, *EMCN*, *LDB2* and *OLFML2A* were exclusively expressed in AF, and thus demonstrated their strong genetic phenotype in AF cells. The gene expression level of NP markers *ARAP2*, *CDKN2B*, *DEFB1*, *DSC3* and *ERFE* was clearly higher in NP compared to AF, even though the NP markers were not exclusively expressed in NP. The absence of AF markers in samples with a clear NP marker profile further proved that these NP samples were free from additional AF tissue, in addition to the IVD scoring. The AF and NP markers could be used stand-alone to simply prove the AF or NP nature of the sample. The total marker expression profile was, however, more conclusive, and a set of both AF and NP markers should be used for a clear classification of disc tissue samples.

Furthermore, the application of both AF and NP markers allowed identification of AF and NP in test samples with undefined disc characteristics (unknown according to IVD scoring). For that, we utilised the marker expression range determined by clear disc samples, meaning the highest and lowest gene expression level measured in AF and NP samples with defined characteristics according to IVD scoring. When the gene expression level of the AF or NP marker was in the range defined by the clear AF and NP samples, respectively, the sample was positive for this marker. The unknown samples with only positive AF marker expression thus clearly originated in the AF compartment of the disc. In contrast, a positive NP marker profile and the lack of AF marker expression proved the NP nature of the unknown sample. Therefore, the marker expression profile revealed the AF or NP character in half of the analysed unknown test samples and thus confirmed the original assignment to either AF or NP compartment of the disc. The novel AF and NP markers were suitable to classify the origin of the disc samples even though the sample contained impurities according to the IVD scoring. However, the overall marker expression profile was not related to any impurity from other tissues adjacently located to the disc, e.g., cartilage endplate, vertebrae or ligamentum flavum. To exclude these impurities, additional markers are necessary that are specific for cartilage, bone or tendon, respectively [69,70,71].

As quantitative gene expression analysis (qPCR) was the preferred assay used later on in quality control of disc tissue samples, all experiments regarding marker expression were performed on mRNA level and thus the protein level was not of interest at this stage of investigation.

According to gene ontology, the novel marker genes are involved in a broad spectrum of biological processes, i.e., signal transduction (*ARAP2*), development (*LDB2*), cell cycle (*CDKN2B*) and biosynthetic processes (*SPTLC3*). Furthermore, genes were mapped to several molecular functions, i.e., transferase activity (*SPTLC3*), kinase activity (*CDKN2B*), catalytic activity (*SPTLC3*), calcium ion binding (*DSC3*, *ADGRL4*), protein binding (*DSC3*), receptor activity (*ADGRL4*) and signal transducer activity (*ADGRL4*). Therefore, it is not surprising that these genes are not exclusively expressed in disc tissue and are also used for several investigations in other tissues. Nevertheless, none of the novel AF and NP markers has been associated before with the intervertebral disc or is regulated in disc degeneration [19,20]. Although *EMCN*, *LDB2* and *OFLM2A* are incidentally listed in two microarray studies, discrimination of AF and NP is not the focus of these studies, but rather discrimination of NP and AC or disc degeneration [10,72]. *EMCN* encodes a mucin-like sialoglycoprotein of the plasma membrane that interferes with the assembly of focal adhesion complexes and inhibits interaction between cells and the ECM [73]. The gene product of *OLFML2A* is localised in the extracellular region and is involved in ECM organisation [74]. *LDB2* has a critical role in cell growth and cell survival, and is highly expressed in different stem cell populations [75]. These novel AF markers could thus be involved in regulation of ECM turnover and maintenance of the AF cell fate. Work by others has shown that *CDKN2B* plays an important role in cell growth inhibition (tumour suppressor genes) and suggests its role in the activation of transforming growth factor (TGF) beta 1 [76]. TGF beta 1 stimulates gene expression of the characteristic ECM molecules aggrecan and collagen type II in pellet culture of NP cells but not in AF cells [77,78]. Hence, we assume a relevant role of the NP marker *CDKN2B* in assembling the ECM of the NP. Nevertheless, less is known about the special function of these genes in the disc and future research is needed to elucidate their role in the disc.

The marker expression profile determined the sample’s fate to be either of AF or NP origin, whereas the IVD score revealed correct tissue assignment and sample purity. This novel quality assessment system can thus be used to select starting material for disc experiments. Samples that have no distinct expression profile of both AF and NP markers and/or did not pass the IVD scoring should be excluded from any AF- or NP-specific experiment. The novel system is also of relevance for quality assurance of cell transplants in disc cell therapy. First, the IVD score and novel markers could prove the disc character of the starting material used for cell isolation and cell expansion. Second, the DD score might be used as a predictor for therapy success as cells from discs with different grades of degeneration have a differential regenerative potential [79,80]. The novel findings could further enable the selection of either AF or NP as starting material and thus provide tissue-targeted cell therapy to repair either AF or NP, respectively. This is of relevance for application in clinics as autologous approaches predominantly utilise herniated disc tissue [81]. The starting material could thus have its origin in either AF or NP, dependent on the hernia location [82,83]. In addition, the markers might also be used to control the cell fate during cell culture and manufacturing process. However, the behaviour of the novel AF and NP markers in cultured disc cells is unknown and further investigations are required.

## 4. Materials and Methods

### 4.1. Human Tissue Material and Tissue Harvesting

Human disc tissue was obtained from patients that underwent mono-, bi- or three-segmental anterior cervical discectomy and fusion surgery. A standard surgical protocol with stepwise removal of tissue from the anterior AF and underlying NP compartment assured correct sample acquisition and thus accurate selection of AF and NP tissue by an experienced surgeon. Tissue samples from separated AF and NP were immediately snap-frozen in liquid nitrogen and stored at −80 °C until further preparation. In total, we obtained AF and NP samples from 28 discs from 17 patients (female/male 9/8) (Table 3) with a mean age of 52 years (range 36–77 years). As almost every disc shows degeneration in humans older than 50 [29], donor age was no exclusion criterion in this study. All discs showed radiographic degenerative changes with grade 3 or 4 according to classification for cervical discs [27].

The study was approved by the ethics committee of the University Medical Center Mannheim (2014-518N-MA, 13 March 2014). All donors provided written informed consent to participate in this study.

### 4.2. Sample Preparation and RNA Isolation

The preparation of frozen AF and NP tissue samples was performed under permanent cooling on dry ice. When macroscopically visible, other tissues like the cartilage endplate, bony endplate or ligamentum flavum were carefully removed. All AF and NP samples first underwent haptic and macroscopic evaluation. Second, a representative cross-section of each sample was taken for histological analysis.

The remaining tissue sample was used for RNA isolation. The frozen tissue was minced and immediately lysed and homogenised in TriReagent (Sigma-Aldrich, Taufkirchen, Germany) using a TissueRuptor (Qiagen, Hilden, Germany). Total RNA was isolated by 1-bromo-3-chloropropane extraction (Sigma-Aldrich) and subsequent purification using the RNeasy Mini Kit including on-column DNase I digestion (Qiagen) according to the manufacturer’s instructions. The RNA quality was determined by measuring RNA integrity number (RIN) value with RNA 6000 Pico Assay using the Bioanalyzer 2100 (Agilent Technologies, Santa Clara, CA, USA). Only samples with RIN > 6.0 were used for analysis.

### 4.3. Histological Analysis

During sample preparation, the tissue was embedded in Tissue-Tek^®^ O.C.T. Compound (Sakura Finetek, Staufen, Germany), frozen in liquid nitrogen and stored at −80 °C until sectioning. Samples were cryosectioned at a thickness of 6 µm.

To show tissue morphology with haematoxylin and eosin (HE) staining, sections were fixed with methanol/acetone (1:1 *v*/*v*) and stained with Mayer’s haematoxylin (Dako, Hamburg, Germany) and eosin-G solution (Carl Roth, Karlsruhe, Germany). Cell nuclei and cartilage-like structures were stained blue or purple, while cytoplasm and non-cartilaginous structures were stained pink.

The extracellular matrix in the samples was analysed using safranin O and alcian blue staining to assess the presence of acid glycosaminoglycans (aGAGs) and sulphated glycosaminoglycans (sGAGs), respectively. For safranin O staining, the sections were fixed with 92% ethanol and stained with 0.7% safranin O solution (Sigma-Aldrich) and subsequently counterstained with 0.2% fast-green solution (Sigma-Aldrich). Structures containing aGAGs were stained red, while others were stained green. For alcian blue staining, the sections were fixed with 4% formaldehyde (Herbeta, Berlin, Germany) and stained with 1% alcian blue 8GS solution (Carl Roth) and counterstained with nuclear fast red–aluminium sulphate solution (Carl Roth). sGAGs were stained blue and cell nuclei were stained pink.

To show calcification with von Kossa staining (dark), sections were fixed with methanol/acetone (1:1 *v*/*v*) and stained in the dark with 5% silver nitrate solution (Sigma-Aldrich) and 5% sodium bicarbonate-formaldehyde solution (Sigma-Aldrich), and subsequently counterstained with nuclear fast red-aluminium sulphate solution (Carl Roth). Calcium deposits were stained black and cell nuclei were stained pink.

### 4.4. Microarray Analysis

Human GeneChip U133 Plus 2.0 (Affymetrix, Santa Clara, CA, USA) was used for genome-wide gene expression profiling covering over 47,000 transcripts (54,765 probes in total including double entries) for AF and NP samples, separately. Samples were prepared with GeneChip^®^ 3′ IVT Express Kit and GeneChip^®^ Hybridisation, Wash and Stain Kit (Affymetrix) according to the manufacturer’s instructions. In brief, 250 ng total RNA was used for cDNA synthesis and subsequent in vitro transcription (IVT) to amplified RNA (aRNA). 12.5 µg fragmented aRNA was used for hybridisation on the chip for 16 h at 45 °C. Finally, the chips were washed, stained and scanned using Affymetrix equipment.

Affymetrix GeneChip Operating Software (GCOS) 1.4 was used to generate CEL data files, for raw data processing and for calculation of signal intensity, signal log ratio (SLR) and *p*-value of pairwise chip comparisons AF/NP. SLR was converted to fold-change (FC) by log2 transformation. From GCOS margin calls for signal presence (detected, not detected) in either AF or NP and margin calls for change in AF/NP comparison (induced, decreased), we calculated percentage frequency of gene expression (detection%) and gene regulation (change%), respectively.

For hierarchical clustering and principle component analysis, we used the R/Bioconductor package affy. Therefore, CEL files underwent Robust Multi-array Average (RMA) normalisation.

### 4.5. Quantitative Gene Expression Analysis

Total RNA was reverse transcribed with the Transcriptor First Strand cDNA Synthesis Kit (Roche, Mannheim, Germany) according to manufacturer’s instructions. The expression of mRNA was evaluated by using quantitative LightCycler^®^ 480 real-time PCR with the Universal Probe Library (UPL) system technology (Roche). Gene expression level was calculated as relative expression between target gene and two reference genes (*ATP5F1B*, *RPL13A*) using the E-method for relative quantification (Roche). Primers and probes for target and reference genes were selected using Roche UPL Assay Design Center and are listed in Table 6. For each sample, measurements were performed in triplicate.

### 4.6. Statistical Analysis

In microarray analysis, signals and FC are shown as mean of all single GeneChips and as mean ± SD of all single pairwise GeneChip comparisons, respectively. Statistical tests regarding signal calls, change calls and *p*-value calculation were performed with GCOS. 

Results in qPCR analysis are shown as mean of the group with an individual data point for each sample. Statistical differences between AF and NP were determined by Mann-Whitney U test using GraphPad Prism 6.0 (GraphPad Software, La Jolla, CA, USA). A *p*-value < 0.05 was considered statistically significant. 

## 5. Conclusions

Although AF and NP tissue from human mature discs showed distinct transcriptomes, the total difference in genome-wide expression was very low. Nevertheless, we identified 11 marker genes that discriminate AF and NP. The specific gene expression of the 5 AF and 6 NP markers proved the correct classification of AF and NP tissue samples. Furthermore, the marker gene expression profile revealed the AF or NP tissue character in unknown samples. In addition, we implemented a novel evaluation system for sample quality, the IVD and DD score. We could thus accurately select clear AF and NP tissue sampled from the mature human disc with low signs of degeneration on the cellular level. As both scorings were designed for small-sized disc samples, they are more meaningful for quality assessment of disc characteristics and disc degeneration in starting material than existing grading systems for total discs. The novel scores and marker genes can test for purity and identity of AF and NP tissue samples. The findings in this study are of high value for quality assurance in preclinical studies or cell-therapeutic approaches used for regenerative treatment of disc diseases.

## 6. Patents

A patent application resulted from the work reported in this manuscript (EP17150489).

## Figures and Tables

**Table 1:** IVD score to assess the character of either AF or NP tissue of small-sized samples.

Estimation of Correct Tissue Type				
	AF		NP	
macroscopic/haptic	dense, annular fibres in multilayer	2	spongy	2
	less defined fibres	1	less spongy	1
	no fibres visible	0	disorganised	0
HE/safranin O staining	well defined half-ring shaped fibres	2	well organised matrix	2
	less defined fibres	1	partly disorganised matrix	1
	no fibres visible	0	loss of nucleus matrix	0
**Correct Expression of Extracellular Matrix Proteins**				
	**AF**		**NP**	
safranin O staining	red dominates, green among lamellae	2	intense red, no green	2
	mixture of red and green	1	reduced red, in parts green	1
	red only, no green	0	faint red, green dominates	0
alcian blue staining	blue dominates, white in lamellae	2	intense blue	2
	reduced blue	1	faint blue	1
	no blue	0	no blue	0
**Evidence of Sample Impurity**				
macroscopic/haptic	not visible			2
	bloody			1
	obviously irregular tissue parts			0
HE/safranin O/alcian blue staining	not visible			2
	tissue structure slightly irregular			1
	any sign of other tissue structure or unusual cell accumulation			0
			maximum IVD score	12

Interpretation: The IVD score contains three subcategories with two scoring items, each graded 0, 1 or 2, resulting in a maximum score of 12 points. Samples resected from AF and NP are scored separately. An IVD score > 6 (without any scoring item scored 0) represents a correctly classified and pure sample of either AF or NP. Otherwise, the sample character is undefined/unknown. AF—annulus fibrosus. HE—haematoxylin-eosin. IVD—intervertebral disc. NP—nucleus pulposus.

**Table 2:** Disc degeneration (DD) score to assess the degenerative grade of small-sized disc samples on cellular level.

macroscopic/haptic	normal and white coloured	0
	slightly harder or yellowish	1
	sclerotic or greyish	2
cellularity (alcian blue staining)	normal, no cell clusters	0
	mixed cellularity, some cell clusters or enlarged cells	1
	mainly clustered cells, chondroid nests present	2
calcification (von Kossa staining)	no sign of calcification	0
	small spots of black staining or extensive greyish staining	1
	intense black staining	2
	maximum DD score	6

Interpretation: The DD score contains three scoring items, each graded 0, 1 or 2 resulting in a maximum score of 6 points. A DD score > 3 (or any scoring item scored 2) represents a sample with severe degeneration.

**Table 3:** Sample information and outcome of IVD and DD scoring of AF and NP tissues sampled from human cervical discs. MRI degeneration grade according to Miyazaki [27].

Donor	Gender	Age	#	Disc	MRI	Scoring AF			Scoring NP		
				Level	Grade	IVD	DD		IVD	DD	
1	m	55	1	C4/5	4	10	0	§	9	1	§
			2	C5/6	4	9	1		7	4 *	+
2	f	43	3	C4/5	3	10	2	§	7	1	§
			4	C5/6	3	5 *	3 *	+	9	2	+
			5	C6/7	3	10	3 *		7	2 *	
3	m	59	6	C4/5	3	6 *	1		4 *	4 *	+
			7	C5/6	3	7	2	§+	3 *	4 *	+
			8	C6/7	3	9	1	+	4 *	3	
4	f	45	9	C5/6	3	11	2	§	9	2	§
5	m	62	10	C3/4	4	2 *	3 *	+	8	2	+
			11	C4/5	4	5 *	1		6	2	§
6	f	76	12	C4/5	3	10	0	§	6 *	3	+
7	m	36	13	C4/5	4	8	1	§	11	1	§
			14	C5/6	4	7 *	3 *	+	9 *	3 *	
			15	C6/7	4	9	3	+	9	2	+
8	m	45	16	C3/4	4	7	4 *	+	7	3 *	+
9	f	76	17	C4/5	4	6 *	4 *	+	7	4 *	
10	f	42	18	C5/6	4	9 *	4 *	+	7	4 *	
			19	C6/7	3	7 *	2		7 *	2	+
11	m	55	20	C4/5	4	2 *	2	+	6 *	1	+
			21	C5/6	4	8	2	§	7	2	§
12	f	64	22	C3/4	4	11	2	§+	5 *	2	
13	f	77	23	C6/7	4	4 *	3 *	§+	6 *	3 *	+
14	m	47	24	C5/6	4	9	2	§+	5 *	1	+
15	f	46	25	C4/5	4	1 *	1	+	7	2	§
16	m	59	26	C5/6	4	9	1		5 *	3 *	+
			27	C6/7	3	8	2	§+	9	2	§
17	f	46	28	C5/6	3	9	2	§+	7	1	§

AF—annulus fibrosus. DD—disc degeneration. IVD—intervertebral disc. MRI—magnetic resonance imaging. NP—nucleus pulposus. # sample number. * any scoring item scored 0 or 2 in IVD or DD score, respectively. § used for microarray analysis. + used for quantitative gene expression analysis.

**Table 4:** Spearman’s correlation analysis of IVD and DD score with donor age and MRI degeneration grade.

Score	Sample Origin	Donor Age		MRI Grade	
		r	*p*-Value	r	*p*-Value
IVD	AF	−0.16	0.424	−0.25	0.196
	NP	−0.57	0.001	−0.07	0.706
DD	AF	−0.25	0.197	+0.13	0.504
	NP	+0.21	0.280	+0.03	0.885

Interpretation: 0–0.19 very weak, 0.2–0.39 weak, 0.4–0.59 moderate, 0.6–0.79 strong, 0.8–1.0 very strong correlation. IVD—intervertebral disc. DD—disc degeneration. MRI—magnetic resonance imaging. r—Spearman’s correlation coefficient.

**Table 5:** AF and NP marker expression profile in unknown test samples.

Sample Origin	#		AF Marker						NP Marker							Marker Match	
			AF1	AF2	AF3	AF4	AF5		NP1	NP2	NP3	NP4	NP5	NP6		AF	NP
AF	4		−	−	NA	−	NA		NA	+	−	−	+	−		0%	40%
AF	10		+	+	NA	+	+		NA	−	−	+	+	−		100%	40%
AF	14		++	+	NA	+	++		NA	−	−	−	−	−		100%	0%
AF	17		−	−	−	−	−		+	−	++	+	+	+		0%	83%
AF	18		−	−	−	+	+		−	−	−	−	+	−		40%	17%
AF	20		+	+	+	+	+		−	−	−	+	−	−		100%	17%
AF	23		+	+	+	++	++		−	−	−	−	−	−		100%	0%
AF	25		++	+	+	+	++		−	−	−	−	−	−		100%	0%
NP	6		−	−	−	−	−		NA	−	−	−	+	−		0%	20%
NP	7		−	−	−	−	−		−	NA	NA	−	NA	−		0%	0%
NP	12		−	−	−	−	−		−	−	−	+	−	+		0%	33%
NP	19		−	−	−	−	NA		++	+	++	+	+	−		0%	83%
NP	20		NA	−	−	NA	−		+	+	−	+	−	−		0%	50%
NP	23		−	−	−	NA	−		−	NA	NA	−	NA	−		0%	0%
NP	24		−	NA	NA	−	−		−	−	+	+	−	−		0%	33%
NP	26		−	−	−	−	−		++	+	−	+	+	+		0%	83%

The test samples were originally sampled from the AF and NP compartment in the disc but showed no clear disc characteristics in IVD scoring (Table 3). # sample number, + gene expression level was within range specified by clear disc tissue samples (Figure 4), ++ higher than range, − lower than range or no expression, NA not available because not enough RNA was available to perform all measurements. AF markers: LDB2 (AF1), ADGRL4 (AF2), EMCN (AF3), ANKRD29 (AF4), OLFML2A (AF5). NP markers: SPTLC3 (NP1), ERFE (NP2), DSC3 (NP3), DEFB1 (NP4), ARAP2 (NP5), CDKN2B (NP6). Marker match is calculated as percentage of positive marker expression of analysed marker genes.

**Table 6:** Primer and probes used in quantitative gene expression analysis.

Gene Symbol	Accession Number #	Primer Sequence		Probe
		Forward	Reverse	
*ADGRL4*	NM_022159.3	tccaaaagaccacagagtttga	atgcagcttttctctttggaa	58
*ANKRD29*	NM_173505.2	gagcaaacatccatgaccaa	ccagcagtaatcgaataacatcc	44
*ARAP2*	NM_015230.3	agacggagtggatgaccagt	cagctggtggccatatatca	61
*CDKN2B*	NM_004936.3	gcggggactagtggagaag	ctgcccatcatcatgacct	17
*DEFB1*	NM_005218.3	tgtctgagatggcctcaggt	gggcaggcagaatagagaca	86
*DSC3*	NM_001941.3	cagaagcacctggagacgat	gagttgttggtagtttgggtca	53
*EMCN*	NM_016242.3 NM_001159694.1	gaactgcttcaagtgaccattct	aacaagtgaattattagctgcctct	29
*ERFE*	NM_001291832.1	ctgctcatctgcatccagtc	gatggtgaagagctcactgct	80
*LDB2*	NM_001290.3 NM_001130834.2 NM_001304434.1 NM_001304435.1	gcctgaagacctgcttgttt	gttgttggttgccttgtgg	53
*OLFML2A*	NM_182487.2	acccccaccaccagtctc	ctcacagctcgcctctctg	19
*SPTLC3*	NM_018327.2	gcagagcttggaaaaagattctc	cagatgcacgatggaacct	22
*ATP5F1B*^1^	NM_001686.3	agaggtcccatcaaaaccaa	tcctgctcaacactcatttcc	50
*RPL13A*^1^	NM_012423.3	caagcggatgaacaccaac	tgtggggcagcatacctc	28

^1^ Housekeeping gene used for relative quantification. # Accession numbers as used for primer design. If different transcript variants existed, all variants are listed here that were covered by the chosen primers.

## Supplementary Materials

Supplementary materials can be found at http://www.mdpi.com/1422-0067/19/6/1761/s1.

## Abbreviations

AC	articular cartilage
ACAN	aggrecan
ATP5F1B	ATP synthase F1 subunit beta
ADGRL4	adhesion G protein-coupled receptor L4
AF	annulus fibrosus
ANKRD29	ankyrin repeat domain 29
ARAP2	ArfGAP with RhoGAP domain, ankyrin repeat and PH domain 2
aRNA	amplified RNA
BCAN	biglycan
BGLAP	bone gamma-carboxyglutamate protein
CA12	carbonic anhydrase 12
CD12	cluster of differentiation 12
cDNA	complementary deoxyribonucleic acid
CDKN2B	cyclin dependent kinase inhibitor 2B
CEP	cartilage endplate
CEL	Affymetrix data file format
COL2A1	collagen type I
COMP	cartilage oligomeric matrix protein
DCN	decorin
DD	disc degeneration
DDD	disc degenerative disease
DEFB1	defensin beta 1
DEG	differentially expressed gene
DSC2	desmocollin 2
DSC3	desmocollin 3
ECM	extracellular matrix
EMCN	endomucin
ERFE	erythroferrin
FC	fold change
FMOD	fibromodulin
FOXF1	forkhead box F1
GCOS	GeneChip Operating Software (Affymetrix)
GLUT-1	glucose transporter 1
GAG	glycosaminoglycan, aGAG and sGAG are acid and sulphated glycosaminoglycans, respectively
HIF1α	hypoxia inducible factor 1 alpha subunit
IVD	intervertebral disc
KRT8	keratin 8
KRT18	keratin 18
KRT19	keratin 19
LDB2	LIM domain binding 2
MRI	magnetic resonance imaging
NC	notochordal
NP	nucleus pulposus
OLFML2A	olfactomedin-like 2A
PCA	principle component analysis
qPCR	quantitative polymerase chain reaction (quantitative gene expression analysis)
RMA	Robust Multi-array Average
RPL13A	ribosomal protein L13a
S100A	S100 calcium binding protein A
SB	subchondral bone
SHH	sonic hedgehog
SLC2A1	solute carrier family 2 member 1
SLR	signal log ratio
SOSTDC1	sclerostin domain containing 1
SPARCL1	SPARC (secreted protein acidic and cysteine rich) like 1
SPTLC3	serine palmitoyltransferase long chain base subunit 3
TEK	TEK receptor tyrosine kinase
TGF	transforming growth factor
TNMD	tenomodulin
UPL	Universal ProbeLibrary (Roche)
VCAN	versican
