# Supplementary material for: Quality Assessment of Surgical Disc Samples Discriminates Human Annulus Fibrosus and Nucleus Pulposus on Tissue and Molecular Level
Source: Int J Mol Sci. 2018 Jun 13;19(6):1761. doi: 10.3390/ijms19061761 (PMC6032144; doi:10.3390/ijms19061761)
Supplement: Supplementary file 1 [file ijms-19-01761-s001.zip › supplement_S1_IJMS_2018-05.pdf]

Supplement Table S1. Microarray data of 267 genes up-regulated in annulus fibrosus (AF) compared to nucleus pulposus (NP). Data from all differently expressed genes with fold-change (FC) AF/NP >2 and change% >80%. Sorting according to FC AF/NP. FC is presented as mean ± SD of all comparisons AF/NP array data with respective p-value (mean). Signal detection is shown as percentage presence (detection%) in the corresponding tissue, AF or NP, and as absolute mean signal value of all AF or NP arrays. Data was obtained from n=11 AF and n=9 NP samples. Gene titles are shown as in annotations of microarray. *actual official NCBI gene symbol and title, old symbol of array annotations is noted in brackets. **more than 2 different gene titles and gene symbols, for all gene titles and symbols see array annotations. /// separates different gene titles and gene symbols for the same gene ID. Selected markers for qPCR are highlighted in bold.

**Gene Title**	**Gene Symbol**	**Gene ID**	**FC AF/NP** mean ± SD	**p-value** mean	**detection%** AF	NP	**Signal** mean AF	NP
**LIM domain binding 2**	**LDB2**	**206481_s_at**	**124,7 ± 7,5**	**0,046**	**91**	**0**	**418**	**2**
selectin E	SELE	206211_at	83,6 ± 9,0	0,055	82	0	376	2
**adhesion G protein-coupled receptor L4**	**ADGRL4* (ELTD1)**	**219134_at**	**73,8 ± 6,7**	**0,025**	**91**	**0**	**422**	**5**
guanylate cyclase 1, soluble, alpha 3	GUCY1A3	227235_at	63,8 ± 7,4	0,020	82	0	316	5
myc target 1	MYCT1	231947_at	59,5 ± 4,9	0,021	91	0	310	4
complement component 7	C7	202992_at	41,3 ± 5,7	0,049	82	0	424	3
CD36 molecule (thrombospondin receptor)	CD36	206488_s_at	40,9 ± 6,9	0,038	100	11	268	24
G protein-coupled receptor 116	GPR116	212951_at	38,7 ± 2,8	0,035	82	0	152	3
von Willebrand factor	VWF	202112_at	38,6 ± 4,0	0,045	91	0	507	11
cysteine/tyrosine-rich 1	CYYR1	228665_at	38,4 ± 3,9	0,054	91	0	279	6
chromosome 8 open reading frame 4	C8orf4	218541_s_at	38,3 ± 4,7	0,042	91	0	149	3
endothelin receptor type B	EDNRB	204273_at	34,9 ± 4,8	0,038	91	22	370	7
GTPase, IMAP family member 7	GIMAP7	228071_at	34,7 ± 7,9	0,072	100	22	345	47
SPARC-like 1 (hevin)	SPARCL1	200795_at	34,0 ± 4,9	0,084	100	89	4263	91
transmembrane 4 L six family member 18	TM4SF18	230061_at	32,7 ± 5,0	0,047	91	22	224	6
**endomucin**	**EMCN**	**222885_at**	**32,3 ± 3,7**	**0,036**	**91**	**0**	**695**	**13**
chemokine (C-X-C motif) ligand 2	CXCL2	209774_x_at	32,1 ± 5,4	0,056	82	0	142	4
hepatocyte growth factor (hepapoietin A; scatter factor)	HGF	209960_at	31,7 ± 4,2	0,021	91	0	47	3
GTPase, IMAP family member 4	GIMAP4	219243_at	30,9 ± 5,8	0,071	82	11	143	27
**ankyrin repeat domain 29**	**ANKRD29**	**229308_at**	**29,0 ± 5,6**	**0,030**	**91**	**0**	**116**	**3**
secreted frizzled-related protein 2	SFRP2	223122_s_at	27,2 ± 9,8	0,118	82	44	1457	50
chemokine (C-X-C motif) ligand 12	CXCL12	209687_at	26,9 ± 6,6	0,054	91	22	1013	41
CD93 molecule	CD93	202878_s_at	24,8 ± 4,0	0,030	100	56	1262	93
**olfactomedin-like 2A**	**OLFML2A**	**213075_at**	**24,4 ± 3,7**	**0,020**	**91**	**0**	**388**	**15**
collagen, type XIV, alpha 1	COL14A1	212865_s_at	24,4 ± 4,1	0,020	100	67	1216	50
cadherin 5, type 2 (vascular endothelium)	CDH5	204677_at	24,2 ± 4,3	0,053	91	0	202	11
early B-cell factor 2	EBF2	244885_at	24,0 ± 5,8	0,046	91	0	135	7
calcitonin receptor-like	CALCRL	234996_at	23,9 ± 5,0	0,051	91	22	221	7
cell adhesion molecule with homology to L1CAM (close homolog of L1)	CHL1	204591_at	23,8 ± 7,1	0,075	91	0	208	5
GTPase, IMAP family member 2	GIMAP2	232024_at	23,2 ± 7,5	0,117	91	22	200	48
platelet/endothelial cell adhesion molecule 1	PECAM1	208982_at	23,1 ± 4,0	0,010	100	33	2355	161

actin, alpha 2, smooth muscle, aorta	ACTA2	200974_at	22,8	±	4,1	0,041	100	67	1259	28
major histocompatibility complex, class II, DR alpha	HLA-DRA	208894_at	22,8	±	9,0	0,081	100	33	1132	143
C-type lectin domain family 14, member A	CLEC14A	226244_at	22,5	±	4,5	0,059	91	0	139	3
regulator of G-protein signaling 5	RGS5	209070_s_at	22,1	±	3,9	0,042	91	0	694	19
RUN domain containing 3B	RUNDC3B	241703_at	20,7	±	4,3	0,047	91	22	130	8
multiple C2 domains, transmembrane 1	MCTP1	235740_at	20,6	±	5,8	0,047	91	22	159	14
chemokine (C-C motif) ligand 14 /// CCL14-CCL15 readthrough /// chemokine (C-C motif) ligand 15	CCL14 /// CCL15…**	205392_s_at	20,5	±	5,1	0,065	91	0	268	10
insulin-like growth factor 1 (somatomedin C)	IGF1	209540_at	20,1	±	3,6	0,012	100	22	525	22
spondin 1, extracellular matrix protein	SPON1	213994_s_at	18,6	±	5,8	0,060	91	11	461	26
GTPase, IMAP family member 1	GIMAP1	1552316_a_at	18,2	±	4,7	0,044	100	11	181	16
microfibrillar associated protein 5	MFAP5	213764_s_at	17,8	±	9,5	0,111	82	22	327	10
platelet endothelial aggregation receptor 1	PEAR1	228618_at	17,6	±	2,9	0,015	91	0	133	5
serum deprivation response	SDPR	222717_at	17,3	±	5,0	0,073	100	33	322	55
interleukin 33	IL33	209821_at	17,1	±	3,0	0,051	91	11	223	10
ribonuclease, RNase A family, 1 (pancreatic)	RNASE1	201785_at	16,9	±	6,9	0,092	91	11	356	34
collagen, type IV, alpha 1	COL4A1	211980_at	16,6	±	3,6	0,005	100	89	2141	85
vestigial like 3 (Drosophila)	VGLL3	227399_at	16,2	±	2,9	0,033	91	22	196	10
neuralized homolog 1B (Drosophila)	NEURL1B	225355_at	15,6	±	3,9	0,031	91	22	470	31
collagen, type XXI, alpha 1	COL21A1	208096_s_at	15,3	±	6,0	0,046	91	0	356	26
serpin peptidase inhibitor, clade F (alpha-2 antiplasmin, pigment epithelium derived factor), member 1	SERPINF1	202283_at	15,2	±	2,9	0,029	91	33	1477	81
tachykinin, precursor 1	TAC1	206552_s_at	15,1	±	4,8	0,061	91	22	181	9
bone morphogenetic protein 5	BMP5	205430_at	15,0	±	3,7	0,051	91	0	208	13
CD200 molecule	CD200	209583_s_at	14,9	±	4,0	0,068	91	44	382	23
podocalyxin-like	PODXL	201578_at	14,7	±	3,5	0,020	100	56	853	71
collagen, type IV, alpha 2	COL4A2	211964_at	14,6	±	3,9	0,016	100	22	698	60
transgelin	TAGLN	205547_s_at	14,5	±	4,1	0,042	91	33	360	17
protein tyrosine phosphatase, receptor type, B	PTPRB	230250_at	14,4	±	3,7	0,064	91	33	429	24
TEK tyrosine kinase, endothelial	TEK	206702_at	13,7	±	4,0	0,048	91	0	157	8
prostate androgen-regulated mucin-like protein 1	PARM1	225809_at	13,6	±	4,0	0,024	91	0	327	20
mannose receptor, C type 1	MRC1	204438_at	13,3	±	4,1	0,054	91	11	198	23
regulator of calcineurin 2	RCAN2	203498_at	13,2	±	2,4	0,001	100	22	408	22
prostaglandin E receptor 4 (subtype EP4)	PTGER4	204897_at	13,1	±	4,2	0,041	100	56	474	52
GTPase, IMAP family member 8	GIMAP8	235306_at	12,9	±	3,8	0,031	100	67	329	32
GTPase, IMAP family member 6	GIMAP6	229367_s_at	12,9	±	3,9	0,031	82	11	285	33
SRY (sex determining region Y)-box 7	SOX7	228698_at	12,8	±	2,7	0,055	100	67	399	21
lysophosphatidic acid receptor 6	LPAR6	218589_at	12,8	±	4,4	0,057	100	33	367	44
guanylate cyclase 1, soluble, beta 3	GUCY1B3	203817_at	12,8	±	4,5	0,036	91	22	397	16
nitric oxide synthase trafficker	NOSTRIN	226992_at	12,3	±	3,7	0,048	82	0	125	11

thrombospondin, type I, domain containing 7A	THSD7A	214920_at	12,2	±	3,9	0,031	100	0	81	7
olfactomedin-like 3	OLFML3	218162_at	12,0	±	2,8	0,001	100	89	577	40
fatty acid binding protein 4, adipocyte	FABP4	203980_at	12,0	±	4,5	0,057	91	44	565	36
CD74 molecule, major histocompatibility complex, class II invariant chain	CD74	209619_at	11,6	±	4,6	0,073	100	56	298	37
sprouty homolog 1, antagonist of FGF signaling (Drosophila)	SPRY1	212558_at	11,5	±	2,7	0,012	100	33	540	39
phosphodiesterase 1A, calmodulin-dependent	PDE1A	208396_s_at	11,4	±	6,1	0,076	91	0	124	8
phosphodiesterase 5A, cGMP-specific	PDE5A	227088_at	10,8	±	2,9	0,022	100	22	293	21
glycoprotein (transmembrane) nmb	GPNMB	201141_at	10,7	±	4,2	0,066	100	100	1814	188
membrane-spanning 4-domains, subfamily A, member 7	MS4A7	223343_at	10,6	±	6,7	0,097	100	22	220	42
arginine vasopressin receptor 1A	AVPR1A	242680_at	10,5	±	3,2	0,036	91	22	185	12
secreted frizzled-related protein 1	SFRP1	202037_s_at	10,4	±	5,8	0,057	91	22	554	33
myosin VIIA and Rab interacting protein	MYRIP	214156_at	9,7	±	4,0	0,029	100	22	154	18
tripartite motif containing 22	TRIM22	213293_s_at	9,6	±	3,2	0,052	100	100	795	162
plasminogen activator, tissue	PLAT	201860_s_at	9,6	±	2,7	0,033	100	89	385	46
matrix metallopeptidase 2	MMP2	201069_at	9,2	±	3,8	0,057	100	89	1272	133
ATP-binding cassette, sub-family A (ABC1), member 8	ABCA8	204719_at	8,9	±	3,1	0,070	100	100	487	40
laminin, alpha 4	LAMA4	202202_s_at	8,8	±	3,6	0,066	100	89	699	65
tetraspanin 7	TSPAN7	202242_at	8,6	±	2,3	0,015	100	56	266	37
caspase 4, apoptosis-related cysteine peptidase pseudogene	LOC643733	240890_at	8,5	±	3,1	0,038	100	44	138	14
ring finger protein 125, E3 ubiquitin protein ligase	RNF125	235199_at	8,4	±	3,5	0,033	91	11	171	27
synaptopodin 2	SYNPO2	225721_at	8,4	±	2,5	0,021	91	22	300	20
multimerin 2	MMRN2	219091_s_at	8,4	±	2,6	0,032	91	67	294	30
cytochrome P450, family 4, subfamily X, polypeptide 1	CYP4X1	227702_at	8,4	±	3,7	0,023	100	56	129	17
family with sequence similarity 198, member B	FAM198B	223204_at	8,3	±	3,2	0,029	100	100	2073	254
follistatin	FST	226847_at	8,3	±	3,8	0,021	91	11	89	13
slit homolog 2 (Drosophila)	SLIT2	228850_s_at	8,1	±	2,5	0,031	100	22	191	20
aquaporin 1 (Colton blood group)	AQP1	209047_at	8,1	±	3,4	0,051	100	100	1772	255
synuclein, alpha interacting protein	SNCAIP	219511_s_at	8,1	±	3,7	0,062	91	0	100	11
ST8 alpha-N-acetyl-neuraminide alpha-2,8-sialyltransferase 4	ST8SIA4	230836_at	8,0	±	4,3	0,084	100	44	204	46
solute carrier organic anion transporter family, member 2A1	SLCO2A1	204368_at	8,0	±	2,6	0,044	91	11	282	24
scavenger receptor class A, member 5 (putative)	SCARA5	229839_at	7,9	±	3,2	0,047	100	33	397	41
CUGBP, Elav-like family member 2	CELF2	202158_s_at	7,8	±	3,5	0,068	100	67	306	57
endothelial cell surface expressed chemotaxis and apoptosis regulator	ECSCR	227780_s_at	7,7	±	3,3	0,051	91	0	216	19
family with sequence similarity 13, member C	FAM13C	1554547_at	7,6	±	3,6	0,067	100	89	330	55
interferon, alpha-inducible protein 27	IFI27	202411_at	7,6	±	2,9	0,042	100	78	507	55
MDS1 and EVI1 complex locus	MECOM	226420_at	7,5	±	3,0	0,051	91	44	134	21
ephrin-B2	EFNB2	202668_at	7,3	±	3,1	0,071	91	22	355	37
secreted frizzled-related protein 4	SFRP4	204052_s_at	7,3	±	3,1	0,042	100	78	391	52
STEAP family member 4	STEAP4	225987_at	7,2	±	3,6	0,070	100	78	759	175

proline rich 16	PRR16	220014_at	6,9 ± 3,3	0,055	91	11	46	6
fibrinogen-like 2	FGL2	204834_at	6,9 ± 3,4	0,078	100	89	292	55
transmembrane and coiled-coil domain family 3	TMCC3	235146_at	6,8 ± 3,2	0,073	100	33	99	26
pleckstrin homology-like domain, family B, member 2	PHLDB2	225688_s_at	6,8 ± 3,5	0,080	100	100	704	92
chloride intracellular channel 2	CLIC2	213415_at	6,7 ± 2,4	0,026	100	56	226	25
interferon-induced protein 44	IFI44	214453_s_at	6,7 ± 3,0	0,053	100	100	186	34
fibulin 1	FBLN1	202995_s_at	6,7 ± 2,4	0,055	100	100	880	93
chromosome 1 open reading frame 115	C1orf115	218546_at	6,7 ± 2,2	0,024	91	33	220	30
A kinase (PRKA) anchor protein 12	AKAP12	227530_at	6,6 ± 2,3	0,039	91	22	212	28
melanoma cell adhesion molecule	MCAM	210869_s_at	6,6 ± 2,9	0,050	100	78	476	32
chemokine (C-C motif) ligand 8	CCL8	214038_at	6,5 ± 4,0	0,052	82	0	201	14
nuclear receptor subfamily 4, group A, member 2	NR4A2	204622_x_at	6,4 ± 2,2	0,005	100	100	967	132
SAM domain and HD domain 1	SAMHD1	234987_at	6,4 ± 2,8	0,052	100	100	690	122
transmembrane protein 119	TMEM119	227300_at	6,3 ± 2,8	0,079	100	78	154	20
major histocompatibility complex, class II, DR beta 1 /// major histocompatibility complex, class II, DR beta 3 ///...**	HLA-DRB1 /// HLA-DRB3…**	215193_x_at	6,3 ± 3,6	0,085	100	100	1046	193
guanylate binding protein 1, interferon-inducible	GBP1	231577_s_at	6,3 ± 3,2	0,050	100	44	152	35
laminin, beta 1	LAMB1	201505_at	6,2 ± 3,3	0,071	100	100	632	102
phosphoglucomutase 5	PGM5	226303_at	6,2 ± 2,7	0,071	91	33	193	18
twist homolog 1 (Drosophila)	TWIST1	213943_at	6,2 ± 3,1	0,066	100	89	478	73
leptin receptor	LEPR	209894_at	6,1 ± 2,2	0,005	100	100	1955	251
protocadherin 17	PCDH17	228863_at	6,1 ± 2,6	0,030	100	56	169	32
complement factor I	CFI	203854_at	6,1 ± 3,9	0,117	100	78	273	43
doublecortin-like kinase 1	DCLK1	229800_at	6,0 ± 3,3	0,064	100	67	212	30
major histocompatibility complex, class II, DR beta 1 /// major histocompatibility complex, class II, DR beta 4 ///...**	HLA-DRB1 /// HLA-DRB4...**	209312_x_at	6,0 ± 3,8	0,089	100	100	1677	341
ATPase, aminophospholipid transporter, class I, type 8B, member 1	ATP8B1	226302_at	5,9 ± 2,5	0,040	100	100	772	116
C1q and tumor necrosis factor related protein 2	C1QTNF2	223749_at	5,9 ± 3,4	0,086	82	67	164	30
proteolipid protein 1	PLP1	210198_s_at	5,9 ± 3,0	0,051	100	11	94	13
activating transcription factor 3	ATF3	202672_s_at	5,7 ± 2,6	0,020	100	89	306	42
complement factor D (adipsin)	CFD	205382_s_at	5,7 ± 2,9	0,057	100	89	930	162
chromosome 4 open reading frame 32	C4orf32	227856_at	5,6 ± 1,8	0,008	100	89	290	54
neuropilin 1	NRP1	212298_at	5,5 ± 2,8	0,067	100	100	538	92
cell adhesion molecule 1	CADM1	209031_at	5,5 ± 2,4	0,048	100	100	893	217
ectonucleoside triphosphate diphosphohydrolase 1	ENTPD1	209473_at	5,5 ± 2,6	0,044	100	78	340	41
early B-cell factor 3	EBF3	227242_s_at	5,5 ± 2,7	0,043	100	33	166	30
phosphatidic acid phosphatase type 2B	PPAP2B	212226_s_at	5,4 ± 2,9	0,078	100	78	447	90
aryl hydrocarbon receptor	AHR	202820_at	5,4 ± 2,7	0,050	100	89	378	103
raftlin, lipid raft linker 1	RFTN1	212646_at	5,3 ± 2,6	0,064	100	56	419	92
dedicator of cytokinesis 4	DOCK4	205003_at	5,3 ± 2,5	0,057	100	100	475	84

coiled-coil domain containing 3	CCDC3	223316_at	5,2 ± 2,1	0,031	100	44	637	125
Rho GTPase activating protein 6	ARHGAP6	206167_s_at	5,1 ± 2,2	0,016	100	89	243	46
nidogen 1	NID1	202007_at	5,1 ± 2,4	0,027	100	100	415	58
uncharacterised LOC100507463	LOC100507463	1555852_at	5,1 ± 3,4	0,115	100	33	99	39
collagen, type XXIV, alpha 1	COL24A1	238732_at	5,0 ± 2,6	0,039	100	22	48	10
tumor necrosis factor receptor superfamily, member 19	TNFRSF19	227812_at	4,9 ± 2,5	0,063	100	67	242	50
laminin, alpha 2	LAMA2	216840_s_at	4,9 ± 2,0	0,016	82	11	136	29
ATPase, aminophospholipid transporter (APLT), class I, type 8A, member 1	ATP8A1	213106_at	4,8 ± 2,2	0,067	100	89	177	42
zinc finger protein, multitype 2	ZFPM2	219778_at	4,7 ± 2,3	0,047	91	33	80	14
aldo-keto reductase family 1, member C3	AKR1C3	209160_at	4,7 ± 2,3	0,038	100	100	508	80
olfactomedin-like 1	OLFML1	217525_at	4,6 ± 2,2	0,066	100	89	167	29
thymocyte selection-associated high mobility group box	TOX	204529_s_at	4,5 ± 2,5	0,048	100	78	101	22
latrophilin 2	LPHN2	206953_s_at	4,5 ± 2,6	0,078	100	100	401	75
FERM domain containing 3	FRMD3	230645_at	4,4 ± 2,0	0,053	91	56	94	17
mesenchyme homeobox 2	MEOX2	206201_s_at	4,4 ± 1,9	0,005	100	100	643	151
carboxypeptidase X (M14 family), member 2	CPXM2	226824_at	4,3 ± 2,1	0,049	91	44	296	68
protein kinase, cGMP-dependent, type I	PRKG1	228396_at	4,3 ± 1,8	0,034	100	89	287	60
cathepsin H	CTSH	202295_s_at	4,2 ± 2,4	0,065	100	67	294	55
nephroblastoma overexpressed	NOV	214321_at	4,2 ± 2,4	0,069	100	100	719	121
C-type lectin domain family 2, member B	CLEC2B	209732_at	4,1 ± 2,6	0,094	100	100	375	115
myristoylated alanine-rich protein kinase C substrate	MARCKS	201670_s_at	4,1 ± 2,5	0,077	100	44	197	56
nuclear receptor subfamily 2, group F, member 2	NR2F2	209120_at	4,0 ± 2,1	0,033	100	100	1387	267
integrin, alpha 9	ITGA9	227297_at	4,0 ± 2,2	0,064	100	100	309	68
frizzled-related protein	FRZB	203697_at	4,0 ± 3,0	0,090	100	100	2105	457
tight junction protein 2 (zona occludens 2)	TJP2	202085_at	4,0 ± 1,9	0,025	100	33	373	86
thrombospondin 1	THBS1	235086_at	4,0 ± 2,3	0,019	100	89	245	52
thrombospondin, type I, domain containing 4	THSD4	222835_at	3,9 ± 2,2	0,073	100	100	302	52
glycine amidinotransferase (L-arginine:glycine amidinotransferase)	GATM	203178_at	3,9 ± 2,7	0,072	100	67	130	37
importin 11 /// leucine rich repeat containing 70	IPO11 /// LRRC70	238488_at	3,9 ± 2,1	0,055	100	78	129	27
actin binding LIM protein 1	ABLIM1	200965_s_at	3,9 ± 2,2	0,054	100	100	655	157
Kruppel-like factor 2 (lung)	KLF2	219371_s_at	3,9 ± 1,7	0,000	100	100	1692	451
multiple EGF-like-domains 6	MEGF6	226869_at	3,5 ± 2,3	0,054	82	0	232	72
nudix (nucleoside diphosphate linked moiety X)-type motif 4 /// nudix-type motif 4 pseudogene 1	NUDT4 /// NUDT4P1	212182_at	3,5 ± 2,4	0,052	100	44	60	20
Rap guanine nucleotide exchange factor (GEF) 5	RAPGEF5	204681_s_at	3,5 ± 3,1	0,129	100	100	378	117
junctional adhesion molecule 2	JAM2	219213_at	3,5 ± 1,7	0,004	100	89	320	72
butyrophilin, subfamily 3, member A2 /// butyrophilin, subfamily 3, member A3	BTN3A2 /// BTN3A3	204820_s_at	3,4 ± 2,2	0,062	100	100	486	165

LIM domain only 2 (rhombotin-like 1)	LMO2	204249_s_at	3,4	±	2,3	0,105	100	100	382	161
Ca++-dependent secretion activator 2	CADPS2	219572_at	3,4	±	1,7	0,025	100	100	219	77
major histocompatibility complex, class II, DP alpha 1	HLA-DPA1	211990_at	3,4	±	2,3	0,089	100	100	1814	503
butyrophilin, subfamily 3, member A2	BTN3A2	209846_s_at	3,3	±	1,9	0,037	100	89	135	35
acyl-CoA synthetase long-chain family member 5	ACSL5	218322_s_at	3,3	±	1,8	0,035	100	56	94	25
EPH receptor A4	EPHA4	227449_at	3,3	±	1,9	0,045	100	100	181	52
Fas (TNF receptor superfamily, member 6)	FAS	204780_s_at	3,3	±	1,9	0,047	100	100	189	57
protein kinase C, eta	PRKCH	218764_at	3,3	±	1,8	0,022	100	100	298	87
cysteine-rich secretory protein LCCL domain containing 1	CRISPLD1	223475_at	3,3	±	2,2	0,042	100	100	1565	529
butyrophilin, subfamily 3, member A3	BTN3A3	204821_at	3,2	±	2,0	0,047	100	78	109	31
early growth response 2	EGR2	205249_at	3,2	±	2,1	0,056	100	89	709	179
platelet-derived growth factor receptor, beta polypeptide	PDGFRB	202273_at	3,2	±	1,7	0,048	100	100	449	107
tropomodulin 2 (neuronal)	TMOD2	226186_at	3,2	±	1,7	0,017	82	0	154	44
suppressor of cytokine signaling 2	SOCS2	203373_at	3,2	±	2,3	0,094	100	100	682	245
transmembrane protein 204	TMEM204	219315_s_at	3,1	±	1,6	0,005	100	100	528	164
apolipoprotein L domain containing 1	APOLD1	221031_s_at	3,1	±	2,2	0,045	100	100	623	177
uncharacterised LOC100287705 /// pleiotrophin	LOC100287705 /// PTN	209465_x_at	3,1	±	2,4	0,122	100	100	401	129
collagen, type XII, alpha 1	COL12A1	225664_at	3,1	±	2,4	0,112	100	100	3138	1059
protein tyrosine phosphatase, receptor type, G	PTPRG	1569323_at	3,1	±	1,6	0,003	100	100	215	58
spectrin repeat containing, nuclear envelope 2	SYNE2	202761_s_at	3,1	±	2,0	0,070	100	100	466	168
activated leukocyte cell adhesion molecule	ALCAM	201952_at	3,0	±	2,0	0,063	100	100	340	116
C1q and tumor necrosis factor related protein 7	C1QTNF7	239349_at	3,0	±	1,9	0,042	100	100	412	127
retinoic acid receptor responder (tazarotene induced) 3	RARRES3	204070_at	3,0	±	1,9	0,065	100	89	145	56
pyruvate dehydrogenase kinase, isozyme 4	PDK4	225207_at	3,0	±	2,4	0,124	100	100	1092	395
integrin, alpha 6	ITGA6	215177_s_at	3,0	±	1,8	0,007	100	100	191	60
microtubule-associated protein 1B	MAP1B	212233_at	3,0	±	1,7	0,022	100	100	663	232
tissue factor pathway inhibitor (lipoprotein-associated coagulation inhibitor)	TFPI	213258_at	3,0	±	2,1	0,106	100	100	482	142
prostaglandin F2 receptor negative regulator	PTGFRN	224937_at	2,9	±	2,2	0,125	100	100	317	102
lysophosphatidylcholine acyltransferase 2	LPCAT2	227889_at	2,9	±	2,0	0,053	100	100	169	66
guanylate binding protein 3	GBP3	223434_at	2,9	±	2,7	0,124	100	100	240	89
Rho guanine nucleotide exchange factor (GEF) 3	ARHGEF3	218501_at	2,9	±	1,7	0,033	100	100	318	111
chromosome 10 open reading frame 116	C10orf116	203571_s_at	2,9	±	1,7	0,018	100	100	1609	514
potassium channel tetramerisation domain containing 12	KCTD12	212192_at	2,9	±	1,8	0,052	100	100	2133	719
ral guanine nucleotide dissociation stimulator-like 1	RGL1	209568_s_at	2,8	±	1,6	0,040	100	100	664	244
XIAP associated factor 1	XAF1	228617_at	2,8	±	1,8	0,040	100	100	501	183
growth arrest-specific 6	GAS6	202177_at	2,8	±	1,7	0,008	100	44	443	151
CD24 molecule	CD24	216379_x_at	2,8	±	2,3	0,111	100	100	643	250
NKF3 kinase family member	PEAK1	225913_at	2,8	±	1,9	0,074	100	67	581	216

endothelial PAS domain protein 1 /// uncharacterised LOC100652809	EPAS1 /// LOC100652809	200878_at	2,8 ± 1,6	0,050	100	100	2390	761
hydroxysteroid (17-beta) dehydrogenase 11	HSD17B11	217989_at	2,8 ± 2,2	0,092	100	100	418	141
insulin-like growth factor binding protein 6	IGFBP6	203851_at	2,8 ± 1,9	0,040	100	100	1685	477
lipoma HMGIC fusion partner	LHFP	218656_s_at	2,7 ± 1,6	0,032	100	100	2503	913
TNF receptor-associated factor 5	TRAF5	204352_at	2,7 ± 1,8	0,066	100	100	208	72
calcium channel, voltage-dependent, alpha 2/delta subunit 1	CACNA2D1	227623_at	2,7 ± 2,0	0,077	100	100	329	127
DEAD (Asp-Glu-Ala-Asp) box polypeptide 60-like	DDX60L	228152_s_at	2,7 ± 2,4	0,121	100	33	224	144
distal-less homeobox 6	DLX6	239309_at	2,7 ± 1,5	0,023	100	67	64	27
family with sequence similarity 176, member C	FAM176C	227188_at	2,7 ± 1,5	0,023	100	100	159	56
frizzled family receptor 4	FZD4	218665_at	2,6 ± 1,6	0,041	100	100	302	125
thrombospondin 4	THBS4	204776_at	2,6 ± 2,2	0,115	100	100	2443	1018
long intergenic non-protein coding RNA 341	LINC00341	219563_at	2,6 ± 1,7	0,025	100	100	897	356
leucine-rich repeat kinase 2	LRRK2	229584_at	2,6 ± 2,6	0,124	100	100	288	155
mitogen-activated protein kinase kinase kinase 1, E3 ubiquitin protein ligase	MAP3K1	225927_at	2,5 ± 1,8	0,088	100	100	449	186
microphthalmia-associated transcription factor	MITF	226066_at	2,5 ± 1,9	0,098	100	100	269	105
DIRAS family, GTP-binding RAS-like 3	DIRAS3	215506_s_at	2,5 ± 1,6	0,072	100	100	146	59
A kinase (PRKA) anchor protein 2 /// PALM2-AKAP2 readthrough	AKAP2 /// PALM2-AKAP2	226694_at	2,5 ± 1,7	0,052	100	100	1663	601
zinc finger protein 36, C3H type, homolog (mouse)	ZFP36	201531_at	2,5 ± 1,7	0,042	100	100	991	386
Kruppel-like factor 11	KLF11	218486_at	2,4 ± 1,6	0,024	100	100	351	136
ribosomal protein S6 kinase, 90kDa, polypeptide 2	RPS6KA2	212912_at	2,4 ± 1,8	0,074	100	100	260	99
estrogen receptor 1	ESR1	205225_at	2,4 ± 1,7	0,048	100	100	365	146
distal-less homeobox 5	DLX5	213707_s_at	2,4 ± 1,5	0,015	100	89	229	117
cytochrome P450, family 1, subfamily B, polypeptide 1	CYP1B1	202437_s_at	2,4 ± 1,9	0,089	100	100	1715	729
heat shock 70kDa protein 1A /// heat shock 70kDa protein 1B	HSPA1A /// HSPA1B	200799_at	2,4 ± 1,4	0,000	100	100	7214	2835
methyltransferase like 7A	METTL7A	207761_s_at	2,4 ± 1,6	0,011	100	100	2700	1083
major histocompatibility complex, class I, E	HLA-E	200904_at	2,3 ± 1,6	0,051	100	100	1349	503
complement factor H /// complement factor H-related 1	CFH /// CFHR1	215388_s_at	2,3 ± 1,9	0,099	100	100	1316	595
long intergenic non-protein coding RNA 597	LINC00597	208109_s_at	2,3 ± 1,6	0,064	100	100	139	54
family with sequence similarity 171, member B	FAM171B	227370_at	2,2 ± 1,7	0,082	100	100	387	174
STAM binding protein-like 1	STAMBPL1	227607_at	2,2 ± 1,6	0,052	100	100	251	114
RNA binding motif, single stranded interacting protein 3	RBMS3	238447_at	2,2 ± 1,7	0,011	100	100	915	374
KIAA1462	KIAA1462	213316_at	2,2 ± 1,6	0,056	100	100	322	149
acetyl-CoA carboxylase beta	ACACB	49452_at	2,1 ± 1,5	0,027	100	100	221	97
homeobox D8	HOXD8	231906_at	2,1 ± 1,6	0,066	100	100	363	204
dishevelled associated activator of morphogenesis 2	DAAM2	212793_at	2,1 ± 1,4	0,012	100	100	530	287

uncharacterised LOC284454	LOC284454	1555847_a_at	2,1	± 1,6	0,090	100	100	346	204
prostaglandin-endoperoxide synthase 1	PTGS1	215813_s_at	2,1	± 1,5	0,073	100	100	314	155
poly (ADP-ribose) polymerase family, member 12	PARP12	218543_s_at	2,1	± 1,5	0,047	100	100	219	107
ankyrin 2, neuronal	ANK2	202920_at	2,1	± 1,8	0,115	100	100	1128	481
runt-related transcription factor 1; translocated to, 1 (cyclin D-related)	RUNX1T1	228827_at	2,1	± 1,5	0,035	100	100	517	251
v-ets erythroblastosis virus E26 oncogene homolog 2 (avian)	ETS2	201328_at	2,1	± 1,5	0,067	100	100	546	234
v-maf musculoaponeurotic fibrosarcoma oncogene homolog F (avian)	MAFF	36711_at	2,1	± 1,4	0,030	100	100	1351	647
CDC14 cell division cycle 14 homolog B (S. cerevisiae)	CDC14B	221556_at	2,1	± 1,4	0,048	100	100	452	207
SAM and SH3 domain containing 1	SASH1	213236_at	2,1	± 1,4	0,003	100	67	561	273
nucleoredoxin	NXN	219489_s_at	2,1	± 1,4	0,033	100	100	411	211
transmembrane protein 30B	TMEM30B	213285_at	2,0	± 1,3	0,020	100	100	488	241
FYVE, RhoGEF and PH domain containing 5	FGD5	226985_at	2,0	± 1,5	0,046	100	100	344	168
