# Supplementary material for: Quality Assessment of Surgical Disc Samples Discriminates Human Annulus Fibrosus and Nucleus Pulposus on Tissue and Molecular Level
Source: Int J Mol Sci. 2018 Jun 13;19(6):1761. doi: 10.3390/ijms19061761 (PMC6032144; doi:10.3390/ijms19061761)
Supplement: Supplementary file 1 [file ijms-19-01761-s001.zip › supplement_S2_IJMS_2018-05.pdf]

Supplement Table S2. Microarray data of 52 genes up-regulated in nucleus pulposus (NP) compared to annulus fibrosus (AF). Data from all differently expressed genes with fold-change (FC) AF/NP <-2 and change% >80%. Sorting according to FC AF/NP. FC is presented as mean ± SD of all comparisons AF/NP array data with respective p-value (mean). Signal detection is shown as percentage presence (detection%) in the corresponding tissue, AF or NP, and as absolute mean signal value of all AF or NP arrays. Data was obtained from n=11 AF and n=9 NP samples. Gene titles are shown as in annotations of microarray. *actual official NCBI gene symbol and title, old symbol of array annotations is noted in brackets. **more than 2 different gene titles and gene symbols, for all gene titles and symbols see array annotations. /// separates different gene titles and gene symbols for the same gene ID. Selected NP markers for qPCR are highlighted in bold.

**Gene Title**	**Gene Symbol***	**Gene ID**	**FC AF/NP**	**p-value**	**detection%**		**signal** mean	
			mean ± SD	mean	**AF**	**NP**	**AF**	**NP**
SRY (sex determining region Y)-box 11	SOX11	204914_s_at	- 5,2 ± 0,4	0,052	91	100	193	765
**serine palmitoyltransferase, long chain base subunit 3**	**SPTLC3**	**227752_at**	**- 4,7 ± 0,5**	**0,028**	**100**	**100**	**91**	**427**
**defensin, beta 1**	**DEFB1**	**210397_at**	**- 4,6 ± 0,4**	**0,077**	**100**	**100**	**434**	**1551**
**desmocollin 3**	**DSC3**	**206033_s_at**	**- 4,4 ± 0,5**	**0,037**	**36**	**100**	**19**	**101**
**erythroferrone**	**ERFE* (FAM132B)**	**229622_at**	**- 3,9 ± 0,4**	**0,039**	**91**	**100**	**234**	**706**
solute carrier family 7 (anionic amino acid transporter light chain, xc- system), member 11	SLC7A11	209921_at	- 3,8 ± 0,4	0,073	100	100	111	315
phosphoserine aminotransferase 1	PSAT1	223062_s_at	- 3,6 ± 0,5	0,050	100	100	349	1075
uncharacterised LOC100652811 /// LOC100653083…**	LOC100652811 /// LOC100653083...**	1553797_a_at	- 3,5 ± 0,5	0,029	100	100	202	562
**ArfGAP with RhoGAP domain, ankyrin repeat and PH domain 2**	**ARAP2**	**213618_at**	**- 3,4 ± 0,4**	**0,083**	**100**	**100**	**115**	**362**
WDNM1-like pseudogene	LOC645638	229566_at	- 3,3 ± 0,4	0,050	45	100	131	345
zinc finger protein 280B	ZNF280B	230789_at	- 3,3 ± 0,5	0,070	45	100	31	107
family with sequence similarity 110, member C	FAM110C	226863_at	- 3,3 ± 0,5	0,033	100	100	171	489
attractin-like 1	ATRNL1	213745_at	- 3,2 ± 0,5	0,096	100	100	209	521
fibronectin 1	FN1	214701_s_at	- 3,2 ± 0,5	0,081	100	100	780	2098
**cyclin-dependent kinase inhibitor 2B (p15, inhibits CDK4)**	**CDKN2B**	**236313_at**	**- 3,1 ± 0,7**	**0,000**	**100**	**100**	**50**	**163**
gamma-aminobutyric acid (GABA) B receptor, 2	GABBR2	209990_s_at	- 3,0 ± 0,4	0,056	64	100	34	126
cytokine receptor-like factor 1	CRLF1	206315_at	- 2,9 ± 0,5	0,071	100	100	528	1373
tumor suppressor candidate 3	TUSC3	213423_x_at	- 2,9 ± 0,6	0,013	100	100	425	1164
UL16 binding protein 2	ULBP2	238542_at	- 2,9 ± 0,5	0,029	91	100	154	366
SPARC related modular calcium binding 1	SMOC1	222783_s_at	- 2,8 ± 0,5	0,072	100	100	339	760
brain and acute leukemia, cytoplasmic	BAALC	218899_s_at	- 2,8 ± 0,5	0,088	100	100	733	1686
ATPase, Na+/K+ transporting, beta 1 polypeptide	ATP1B1	201242_s_at	- 2,8 ± 0,5	0,046	100	100	634	1528
SLIT and NTRK-like family, member 4	SLITRK4	232636_at	- 2,8 ± 0,6	0,042	100	100	181	484
calcyphosine	CAPS	231729_s_at	- 2,7 ± 0,6	0,028	100	100	225	586
STEAP family member 3, metalloreductase	STEAP3	1554830_a_at	- 2,7 ± 0,5	0,080	100	100	92	223
protein phosphatase 1, regulatory (inhibitor) subunit 14C	PPP1R14C	226907_at	- 2,6 ± 0,5	0,089	100	100	362	861
cadherin 19, type 2	CDH19	206898_at	- 2,6 ± 0,6	0,021	100	100	729	1827
desmoglein 2	DSG2	217901_at	- 2,5 ± 0,6	0,019	100	100	740	1756
tumor necrosis factor, alpha-induced protein 6	TNFAIP6	206026_s_at	- 2,5 ± 0,5	0,087	100	100	3483	7615
desmocollin 2	DSC2	226817_at	- 2,5 ± 0,5	0,081	100	100	143	311

ADAM metallopeptidase with thrombospondin type 1 motif, 6	ADAMTS6	237411_at	- 2,4 ± 0,6	0,064	100	100	670	1569
family with sequence similarity 167, member A	FAM167A	226614_s_at	- 2,4 ± 0,6	0,075	100	100	78	172
H19, imprinted maternally expressed transcript (non-protein coding) /// microRNA 675	H19 /// MIR675	224997_x_at	- 2,4 ± 0,6	0,059	100	100	370	846
UDP-N-acetyl-alpha-D-galactosamine:polypeptide N-acetylgalactosaminyltransferase 7 (GalNAc-T7)	GALNT7	222587_s_at	- 2,3 ± 0,6	0,039	100	100	230	534
DnaJ (Hsp40) homolog, subfamily C, member 12	DNAJC12	223721_s_at	- 2,3 ± 0,6	0,040	100	100	71	164
uncharacterised LOC100289058	LOC100289058	1563077_at	- 2,2 ± 0,6	0,080	100	100	260	594
coiled-coil domain containing 80	CCDC80	243864_at	- 2,2 ± 0,6	0,082	100	100	391	768
retinoic acid receptor, beta	RARB	205080_at	- 2,2 ± 0,6	0,066	100	100	455	1065
melanoma antigen family D, 4 /// melanoma antigen family D, 4B…**	MAGED4 /// MAGED4B…**	221261_x_at	- 2,2 ± 0,6	0,056	100	100	147	299
long intergenic non-protein coding RNA 86 /// long intergenic non-protein coding RNA 87	LINC00086 /// LINC00087	227909_at	- 2,1 ± 0,5	0,110	100	100	695	1350
delta/notch-like EGF repeat containing	DNER	226281_at	- 2,1 ± 0,5	0,090	100	100	3059	5751
dermatopontin	DPT	207977_s_at	- 2,1 ± 0,6	0,096	100	100	1168	2400
receptor tyrosine kinase-like orphan receptor 1	ROR1	232060_at	- 2,1 ± 0,6	0,076	100	100	296	569
isthmin 1 homolog (zebrafish)	ISM1	235182_at	- 2,1 ± 0,6	0,027	100	100	1784	3579
WNT1 inducible signaling pathway protein 3	WISP3	210861_s_at	- 2,1 ± 0,6	0,066	100	100	597	1267
phospholipase A2, group IVA (cytosolic, calcium-dependent)	PLA2G4A	210145_at	- 2,1 ± 0,6	0,066	100	100	998	2127
fibronectin type III domain containing 3B	FNDC3B	222693_at	- 2,1 ± 0,7	0,047	100	100	350	679
immunoglobulin superfamily, member 3	IGSF3	202421_at	- 2,1 ± 0,7	0,014	100	100	245	513
asparagine synthetase (glutamine-hydrolyzing)	ASNS	205047_s_at	- 2,1 ± 0,6	0,056	100	100	317	667
mucolipin 3	MCOLN3	220484_at	- 2,0 ± 0,6	0,089	100	100	183	330
ligand of numb-protein X 1, E3 ubiquitin protein ligase	LNX1	223611_s_at	- 2,0 ± 0,7	0,075	100	100	615	1179
potassium voltage-gated channel, KQT-like subfamily, member 5	KCNQ5	244623_at	- 2,0 ± 0,6	0,075	100	100	457	911
