# Supplementary material for: Quality Assessment of Surgical Disc Samples Discriminates Human Annulus Fibrosus and Nucleus Pulposus on Tissue and Molecular Level
Source: Int J Mol Sci. 2018 Jun 13;19(6):1761. doi: 10.3390/ijms19061761 (PMC6032144; doi:10.3390/ijms19061761)
Supplement: Supplementary file 1 [file ijms-19-01761-s001.zip › supplement_S3_IJMS_2018-05.pdf]

Supplement Table S3. Microarray data of previously identified disc-related genes. Gene titles are shown as in array annotations (common alias in brackets). Sorting in each category according to fold change (FC) AF/NP. FC is presented as mean ± SD of all comparisons AF/NP array data with respective p-value (mean). Change% describes the up- and down-regulation in AF tissue and is shown as percentage of all comparisons AF/NP. Signal detection is shown as percentage presence (detection%) in the corresponding tissue, AF or NP, and as absolute mean signal value of all AF or NP arrays. Array data were obtained from n=11 AF and n=9 NP samples. AF - annulus fibrosus. NP - nucleus pulposus. Genes are highlighted in bold, when this gene was differentially expressed (FC at least 2 and change >80%) and regulation was in line with literature.

**Gene Title**	**Gene Symbol**	**FC AF/NP**			**p-value**	**change% in AF**		**detection%**		**signal** mean	
		mean	±	SD	mean	up	down	**AF**	**NP**	**AF**	**NP**
**Genes previously reported with higher expression in NP compared to AF**											
human disc											
C-type lectin domain family 2, member B	CLEC2B	4,0	±	2,6	0,095	86	4	100	100	375	115
alpha-2-macroglobulin	A2M	2,2	±	2,3	0,193	72	9	100	100	2562	1595
cadherin 2, type 1, N-cadherin	CDH2	1,5	±	3,3	0,466	24	10	45	22	86	58
carbonic anhydrase XII	CA12	1,4	±	1,6	0,256	21	1	82	56	129	85
TYRO3 protein tyrosine kinase	TYRO3	-1,5	±	0,7	0,193	1	39	0	22	58	86
neural cell adhesion molecule 1	NCAM1	-1,5	±	0,3	0,336	0	1	0	0	12	16
**desmocollin 2**	**DSC2**	**-2,5**	**±**	**0,5**	**0,081**	**3**	**81**	**100**	**100**	**143**	**311**
sarcoglycan, gamma	SGCG	-2,5	±	0,3	0,179	6	69	27	78	28	82
non-human disc											
sclerostin domain containing 1	SOSTDC1	3,8	±	4,1	0,188	6	0	0	0	14	3
CD24 molecule	CD24	2,8	±	2,3	0,111	81	6	100	100	643	250
keratin 18	KRT18	1,2	±	1,2	0,226	6	1	100	89	163	133
keratin 8	KRT8	-1,0	±	0,7	0,435	0	2	91	56	44	49
keratin 19	KRT19	-1,0	±	0,7	0,544	0	1	0	0	2	2
versican	VCAN	-1,4	±	0,7	0,186	5	60	100	100	3024	4483
solute carrier family 2, member 1 (GLUT-1)	SLC2A1	-1,6	±	0,7	0,119	2	73	100	100	194	304
**Genes previously reported with higher expression in AF compared to NP**											
human disc											
**SPARC-like 1 (hevin)**	**SPARCL1**	**33,6**	**±**	**4,9**	**0,084**	**91**	**6**	**100**	**89**	**4263**	**91**
nuclear factor of activated T-cells, cytoplasmic, 4	NFATC4	1,9	±	2,1	0,114	41	0	45	11	91	51
non-human disc											
**secreted frizzled-related protein 2**	**SFRP2**	**27,2**	**±**	**9,8**	**0,118**	**82**	**9**	**82**	**44**	**1457**	**50**
**serpin peptidase inhibitor, clade F, member 1**	**SERPINF1**	**15,2**	**±**	**2,9**	**0,029**	**94**	**2**	**91**	**33**	**1477**	**81**
tenomodulin	TNMD	7,6	±	5,8	0,142	53	0	64	0	88	5
regulator of G-protein signaling 5	RGS5	7,5	±	3,2	0,034	66	0	82	0	406	20
keratocan	KERA	3,2	±	2,0	0,035	79	0	100	33	95	23
**collagen, type XII, alpha 1**	**COL12A1**	**3,1**	**±**	**2,4**	**0,112**	**85**	**8**	**100**	**100**	**3138**	**1059**

myosin, heavy chain 11, smooth muscle	MYH11	2,7	±	3,8	0,278	21	0	36	0	36	12
ADAM metallopeptidase with thrombospondin type 1 mot 17	ADAMTS17	-1,2	±	0,6	0,409	12	28	100	100	120	151
collagen, type V, alpha 1	COL5A1	-1,4	±	0,6	0,297	17	55	100	100	2392	2852
**Other genes related to the disc**											
collagen type I	COL1A1	3,8	±	3,6	0,184	67	11	100	100	1645	390
hemoglobin, beta	HBB	2,3	±	2,7	0,283	57	11	100	100	10560	6670
thrombospondin 2 (TSP-2)	THBS2	1,8	±	1,6	0,122	67	4	100	100	2468	1490
decorin	DCN	-1,1	±	0,8	0,307	9	34	100	100	11028	12611
biglycan	BGN	-1,1	±	0,8	0,349	6	28	100	100	3886	4448
fibromodulin	FMOD	-1,1	±	0,8	0,306	3	39	100	100	9649	11057
hypoxia inducible factor 1, alpha subunit	HIF1A	-1,1	±	0,8	0,285	0	38	100	100	6217	7092
collagen type II	COL2A1	-1,2	±	0,7	0,358	15	43	100	100	6346	7342
aggrecan	ACAN	-1,2	±	0,6	0,434	26	42	100	100	5451	6291
forkhead box F1	FOXF1	-1,2	±	0,7	0,317	9	29	100	100	550	641
ovostatin 2	OVOS2	-1,4	±	0,5	0,328	21	47	100	100	272	350
proteoglycan 4	PRG4	-1,7	±	0,7	0,072	1	79	100	100	7113	12012
paired box 1	PAX1	-1,9	±	0,7	0,059	0	77	100	100	297	534
**Genes related to disc development or progenitor cells**											
TEK tyrosine kinase, endothelial (Tie2)	TEK	13,5	±	4,0	0,048	89	0	91	0	157	8
sonic hedgehog	SHH	1,1	±	3,4	0,498	0	0	0	0	13	12
T, brachyury homolog	T	-1,1	±	0,5	0,536	0	2	0	0	7	9
